# Supplementary material for: Double Deprotonation of CH3CN by an Iron‐Aluminium Complex
Source: Angew Chem Weinheim Bergstr Ger. 2023 Mar 9;135(16):e202219212. doi: 10.1002/ange.202219212 (PMC10952947; doi:10.1002/ange.202219212)
Supplement: Supplementary file 4 — Supporting Information [file ANGE-135-0-s001.pdf]

Angewandte Chemie
Eine Zeitschrift der Gesellschaft Deutscher Chemiker

Supporting Information

Double Deprotonation of CH_3CN by an Iron-Aluminium Complex

B. Stadler, N. Gorgas, A. J. P. White, M. R. Crimmin**

1 General experimental 2
2 Synthetic procedures 3
3 NMR monitoring studies 11
4 X-Ray data 13
5 Computational methods 15
6 NMR spectra of isolated compounds 32
7 References 48

1 General experimental

All manipulations were carried out using standard Schlenk-line and glovebox techniques under an inert atmosphere of argon or dinitrogen. A MBraun Labmaster glovebox was employed, operating at <0.1 ppm O_2 and <0.1 ppm H_2O. Solvents were dried over activated alumina from a SPS (solvent purification system) based upon the Grubbs design, stored over activated 4 Å molecular sieves and degassed before use. Glassware was dried for 12 h at 120 °C prior to use. C_6D_6 was dried over 4 Å molecular sieves and freeze-pump-thaw degassed thrice before use. Chemicals were purchased from Sigma Aldrich, Fluorochem, Alfa Aesar, and VWR. $^{Xyl}BDIAlH_2$[1] (^{R}BDI = {(RNCMe}$_2$CH), Xyl = 2,6–Me–C_6H_4), and **1a**[2] were synthesised according to literature procedures. NMR Spectra were recorded on Bruker 400 MHz or 500 MHz at 298 K unless otherwise stated and values recorded in ppm. Data were processed in MestReNova software. Where needed, chemical shifts were assigned with the assistance of 2D NMR (HSQC, HMBC, COSY) spectra. IR spectra were recorded on an Agilent Cary630 ATR FTIR spectrometer. Elemental analyses were performed by Elemental Labs (https://www.elementallab.co.uk/).

2 Synthetic procedures

2.1 Synthesis of $(PMe_3)_3FeH_2Br_2Al^{Xyl}BDI$

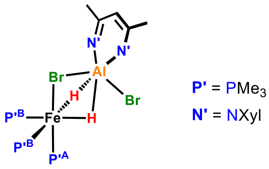

In a glovebox, a Schlenk flask was charged with to $FeBr_2$ (324 mg, 1.50 mmol, 1.0 equiv.) and a magnetic stirrer bar. A solution of PMe_3 (495 µL, 4.80 mol, 3.2 equiv.) in toluene (ca. 10 ml) was added, which resulted in the immediate formation of a deep purple solution. Then a solution of $^{Xyl}BDIAlH_2$ (501 mg, 1.50 mmol, 1.0 equiv.) in toluene (ca. 10 mL) was added, and the resulting mixture was stirred at room temperature for 30 mins, by when a colour change to red was observed. The volatiles were removed under reduced pressure and the solid washed with *n*-pentane (3 x 10 mL). The resulting solid was dried *in vacuo* to yield $(PMe_3)_3FeH_2Br_2Al^{Xyl}BDI$ as a salmon-coloured free flowing powder (821 mg, 1.06 mmol, 70 %).

^{1}H NMR (400 MHz, C_6D_6, 298 K): δ 7.06-6.97 (m, 6H, Xyl C*H*) 5.45 (s, 1H, BDI C*H*), 3.11 (s, 6H, Xyl C-CH_3), 2.50 (s, 6H, Xyl C-CH_3), 1.58 (s, 6H, BDI C-CH_3), 1.26 (br, 9H, $P^A(CH_3)_3$, FWHM = 109 Hz), 0.81 (bs, 18H, $P^B(CH_3)_3$, -13.04 (br, 2H, Fe-µH_2-Al, FWHM = 85.5 Hz).

^{31}P{^{1}H} NMR (162 MHz, C_6D_6, 298 K): δ 47.6 (t, 1P, P^ACH_3, $^2J_{P-P}$ = 44.7 Hz), 16.7 (d, 2P, P^BMe_3, $^2J_{P-P}$ = 44.7 Hz),

^{13}C{^{1}H} NMR (101 MHz, C_6D_6, 298 K): δ 169.5 (2C, BDI *C*N), 147.1 (2C, Xyl *C*N), 136.7 (2C, Xyl *CCH$_3$*), 136.2 (2C, Xyl *C*CH$_3$), 129.0 (2C, Xyl *C*H), 128.8 (2C, Xyl *C*H), 125.4 (2C, Xyl *C*H), 101.4 (BDI *C*H), 24.5 (2C, BDI *C*H$_3$), 23.0 (2C, Xyl *C*H$_3$), 22.2 (m, 9C, $P^{AB}(CH_3)_3$, 20.18 (2C, Xyl *C*H$_3$).

EA calc for $C_{30}H_{54}AlBr_2FeN_2P_3$ ($(PMe_3)_3FeH_2Br_2Al^{Xyl}BDI$): C 46.30 %, H 6.99 %, N 3.60 %. Calc for $C_{37}H_{62}AlBr_2FeN_2P_3$ ($(PMe_3)_3FeH_2Br_2Al^{Xyl}BDI$.toluene): C 51.05 %, H 7.18 %, N 3.22 %. Found C 50.54 %, H 7.17 %, N 3.21 %.

2.2 Synthesis of **1b**

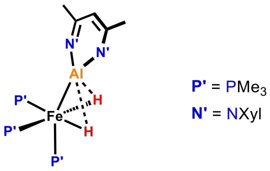

In a glovebox a Schlenk flask was charged with Mg turnings (50 mg, 2 mol, 3.3 equiv.) that have been slightly crushed with a spatula. To this was added a solution of $(PMe_3)_3FeH_2Br_2Al^{Xyl}$BDI (482 mg, 0.619 mmol, 1 equiv.) in THF (10 mL) and the mixture was stirred at room temperature. After 2 hours, the colour had completely changed from red orange to deep red-brown. The solids were filtered via cannula, and the volatiles removed under reduced pressure. The resulting dark solid was extracted with *n*-hexane. The combined extracts were filtered, and the volatiles removed *in vacuo* to yield **1b** as a dark red-brown free flowing powder (312 mg, 0.504 mmol, 81 %).

^{1}H NMR (400 MHz, C_6D_6, 298 K): δ 7.07-6.99 (m, 6H Xyl C*H*), 4.92 (s, 1H, BDI C*H*), 2.34 (s, 12H, Xyl CH_3), 1.31 (s, 6H, BDI CH_3), 1.14 (bs, 27H, P CH_3), -16.33 (q, 2H, Fe-μH_2-Al, $^2J_{P-H}$ = 20.8 Hz)

^{31}P{^{1}H} NMR (162 MHz, C_6D_6, 298 K): δ 26.2 (s, 3P)

^{13}C{^{1}H} NMR (101 MHz, C_6D_6, 298 K): δ 167.6 (2C, BDI *C*N), 147.2 (2C, Xyl *C*N), 134.0 (4C, Xyl *C*CH_3), 129.4 (4C, Xyl *C*H), 126.2 (2C, Xyl *C*H), 99.6 (1C, BDI *C*H), 30.4 (m, 9C, P(CH_3)$_3$), 23.9 (2C, BDI CH_3), 19.9 (4C, Xyl CH_3)

IR (ATR, solid, ν/cm^{-1}): 1769 (w, Fe-μH_2-Al), 1644 (w, Fe-μH_2-Al)

EA calc for $C_{30}H_{54}AlFeN_2P_3$ (2a): C 58.26 %, H 8.80 %, N 4.53 %. Found C 58.11 %, H 8.62 %, N4.54 %.

2.3 Synthesis of **2a**

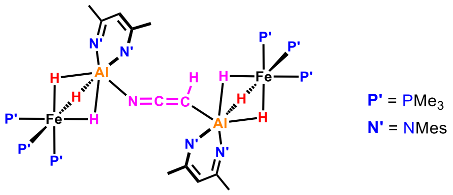

Method 1: In a glovebox, in a J. Youngs NMR tube[1] was charged with a solution of **1a** (15 mg, 0.0231 mmol, 1 equiv.) in C_6D_6 (0.5 mL) MeCN (1.2 µL, 0.0231 mmol, 1 equiv.[2]) was added. NMR tube was inverted 10 times, and over the course of the first 10 minutes, a colour change to orange-brown was observed. The mixture was left at rt. for 3 days, by when the solution turned orange and orange single crystals of **2a.C$_6$D$_6$** suitable for X-ray crystallography formed in the NMR tube. The supernatant was decanted, and the crystals were washed with *n*-pentane (3 x 1 mL), then dried *in vacuo* yielding **2a.C$_6$D$_6$** as a highly insoluble orange crystalline solid (5 mg, 0.00375 mmol, 33 %).

Method 2: In a glovebox, a J. Youngs NMR tube was charged with a solution of **1a** (5 mg, 0.0077 mmol, 1 equiv.) in C_6D_6 (0.5 mL) and MeCN (3.75 µL, 0.0718 mmol, 9.3 equiv.). The NMR tube was inverted 10 times, resulting in an immediate colour change to light yellowish brown. After 10 minutes, the volatiles were removed *in vacuo* yielding a yellow-brown residue. A solution of **1a** (5 mg, 0.0077 mmol, 1 equiv.) in C_6D_6 (0.5 mL) was added, and the NMR tube inverted 10 times. After 7 days, the orange solution was decanted the orange microcrystalline solid was washed with pentane (3 x 1 mL), and dried *in vacuo* to yield **2a.C$_6$D$_6$** (3 mg, 0.00225, 29 %) as a highly insoluble orange crystalline powder.

^{1}H NMR (500 MHz, THF-d$_8$, 298 K): δ 6.85 (s, 2H, Mes C*H*), 6.79 (s, 2H, Mes C*H*), 6.75 (s, 2H, Mes C*H*), 6.70 (s, 2H, Mes C*H*), 5.16 (s, 1H, BDI C*H*), 5.10 (s, 1H, BDI C*H*), 2.69 (s, 6H, Mes C*H*$_3$), 2.59 (s, 6H, Mes C*H*$_3$), 2.24 (s, 6H, Mes C*H*$_3$), 2.21 (s, 6H, Mes C*H*$_3$), 2.19 (s, 6H, Mes C*H*$_3$), 2.18 (s, 6H, Mes C*H*$_3$), 1.53 (s, 6H, BDI C*H*$_3$), 1.51 (s, 6H, BDI C*H*$_3$), 0.91 (s, 54H, P(C*H*$_3$)$_3$), 0.25 (s, 1H, C*H*CN), -15.79 (br q, 3H, Fe-µ*H*$_3$-Al), -16.05 (br s, 3H, Fe-µ*H*$_3$-Al).

^{1}H NMR (400 MHz, C_6D_6, 298 K): δ 7.03 (s, 2H, Mes C*H*), 6.96 (s, 2H, Mes C*H*), 6.86 (s, 2H, Mes C*H*), 6.79 (s, 2H, Mes C*H*), 5.28 (s, 1H, BDI C*H*), 5.15 (s, 1H, BDI C*H*), 3.01 (s, 6H, Mes C*H*$_3$), 2.84 (s, 6H, Mes C*H*$_3$), 2.54 (s, 6H, Mes C*H*$_3$), 2.35 (s, 6H, Mes C*H*$_3$), 2.28 (s, 6H, Mes C*H*$_3$), 2.25 (s, 6H, Mes C*H*$_3$), 1.76 (s, 6H, BDI C*H*$_3$), 1.59 (s, 6H, BDI C*H*$_3$), 1.08 (s, 27H, P(C*H*$_3$)$_3$), 0.98 (s, 27H, P(C*H*$_3$)$_3$), 0.56 (s, 1H, C*H*CN), -15.54 (bs, 3H, Fe-µ*H*$_3$-Al), -15.85 (bs, 3H, Fe-µ*H*$_3$-Al).

^{31}P{^{1}H} NMR (202 MHz, THF-d$_8$, 298 K): δ 29.3 (s, 3P), 29.1 (s, 3P).

^{31}P{^{1}H} NMR (162 MHz, C_6D_6, 298 K): δ 29.6 (s, 3P), 29.3 (s, 3P).

^{13}C{^{1}H} NMR (101 MHz, THF-d$_8$, 298 K): δ 167.1 (2C, BDI *C*N), 166.8 (2C, BDI *C*N), 148.1 (2C, Mes *C*N), 147.0 (2C, Mes *C*N), 137.7 (2C, Mes *C*CH$_3$), 137.5 (2C, Mes *C*CH$_3$), 134.9 (2C, Mes *C*CH$_3$), 134.2 (2C, Mes *C*CH$_3$), 134.2 (2C, Mes *C*CH$_3$), 134.1 (2C, Mes *C*CH$_3$), 130.6 (2C, Mes *C*H), 130.3 (2C, Mes *C*H), 129.5 (2C, Mes *C*H), 129.4 (2C, Mes *C*H), 101.3 (1C, BDI *C*H), 100.4 (1C, BDI *C*H), 27.0 (18C, P-*C*H$_3$), 24.7 (2C, BDI *C*H$_3$), 24.4 (2C, BDI *C*H$_3$), 22.4 (2C, Mes *C*H$_3$), 21.6 (4C, Mes *C*H$_3$), 21.1 (2C, Mes *C*H$_3$),

[1] The choice of reaction vessel as well as minimal agitation after the initial mixing is important, stirring in a vial for 72 h gives only trace **2a**.

[2] Using the theoretically optimal, 0.5 equiv. of CH_3CN results in the formation of only trace **2a**, and forms mainly unidentified side-products instead.

20.8 (4C, Mes $\underline{C}H_3$), 20.4 (2C, Mes $\underline{C}H_3$). The ^{13}C resonances in the $[CHCN]^{2-}$ fragment were not observed.

IR (ATR, solid, ν/cm^{-1}): 2029 (m, C=N), 1750 (w, Fe-μ$\underline{H}_2$-Al), 1719 (w, Fe-μ$\underline{H}_2$-Al)

EA: due to the thermal instability of this compound CHN analysis was not obtained.

2.4 Synthesis of **2b**

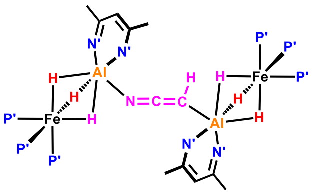

In a glovebox, a 20 mL vial was charged with a solution of **1b** (600 mg, 0.97 mmol, 1 equiv.) in toluene (6 mL) and MeCN (24 µL, 0.46 mmol, 0.47 equiv.) was added. The mixture was stirred at 25 °C for 24 h, by which point a large amount orange solid formed. The volatiles were removed under vacuum, the solid was washed with pentane (4 x 5 mL) and dried under vacuum to yield **2b.toluene** as an orange solid (423 mg, 68 % yield). Crystals suitable for X-ray diffraction as well as samples of analytical purity could be grown from a concentrated solution of **2b** in a 1:1 Et_2O:toluene mixture at -35 °C over several weeks.

2b-[^{13}C] was synthesised using **1b** (60 mg, 0.097 mmol, 1 equiv.) and MeCN-[2-^{13}C] (2.4 µL, 0.046 mmol, 0.47 equiv.) using an analogous procedure.

^{1}H NMR (500 MHz, C_6D_6, 298 K): δ 7.32 (m, 1H), 7.21 (t, J = 4.6 Hz, 2H), 7.09 – 7.01 (m, 5H), 7.01 (d, J = 7.5 Hz, 2H), 6.99 – 6.96 (m, 2H), 5.26 (s, 1H, BDI-C$\underline{H}$), 5.13 (s, 1H, BDI-C$\underline{H}$), 3.02 (s, 6H, Xyl C$\underline{H}_3$), 2.86 (s, 6H, Xyl C$\underline{H}_3$), 2.54 (s, 6H, Xyl C$\underline{H}_3$), 2.35 (s, 6H, Xyl C$\underline{H}_3$), 1.70 (s, 6H, BDI CC$\underline{H}_3$), 1.54 (s, 6H, BDI CC$\underline{H}_3$), 1.05 (br q, $^2J_{H-P}$ = 1.9 Hz, 27H, P(C$\underline{H}_3$)$_3$), 0.96 (br q, $^2J_{H-P}$ = 2.0 Hz, 27H, P(C$\underline{H}_3$)$_3$), 0.56 (s, 1H, C$\underline{H}$CN), -15.54 (br q, 3H, Fe-µ$\underline{H}_3$-Al), -15.87 (br s, 3H, Fe-µ$\underline{H}_3$-Al).

^{31}P{^{1}H} NMR (202 MHz, C_6D_6, 298 K) δ 29.6 (s, 3P), 29.3 (s, 3P).

^{13}C NMR (126 MHz, C_6D_6, 298 K): δ 166.4 (2C, BDI $\underline{C}$N), 166.2 (2C, BDI $\underline{C}$N), 150.2 (2C, Xyl $\underline{C}$N), 149.0 (2C, Xyl $\underline{C}$N), 137.6 (2C, Xyl $\underline{C}$CH$_3$), 137.3 (2C, Xyl $\underline{C}$CH$_3$), 134.2 (2C, Xyl $\underline{C}$CH$_3$), 134.0 (2C, Xyl $\underline{C}$CH$_3$), 129.5 (2C, Xyl $\underline{C}$H), 129.2 (2C, Xyl $\underline{C}$H), 128.6 (2C, Xyl $\underline{C}$H), 128.4 (2C,Xyl $\underline{C}$H), 125.3 (2C, Xyl $\underline{C}$H), 124.9 (2C, Xyl $\underline{C}$H), 101.0 (1C, BDI $\underline{C}$H), 100.0 (1C, BDI $\underline{C}$H), 27.0 – 26.7 (m, 9C, P($\underline{C}$H$_3$)$_3$), 26.8 – 26.4 (m, 9C, P($\underline{C}$H$_3$)$_3$), 24.6 (2C, BDI C$\underline{C}$H$_3$), 24.2 (2C, BDI CC$\underline{H}_3$), 22.0 (2C, Xyl C$\underline{C}$H$_3$), 21.1 (2C, Xyl C$\underline{C}$H$_3$), 20.7 (2C, Xyl C$\underline{C}$H$_3$), 20.1 (2C, Xyl C$\underline{C}$H$_3$). The ^{13}C resonances of the [CHCN]$^{2-}$ fragment are not observable using ^{13}C{^{1}H}, ^{1}H-^{13}C HMBC, and ^{1}H-^{13}C HSQC experiments.

IR (ATR, solid, ν/cm^{-1}): 2038 (m, C=N), 1770 (w, Fe-µ$\underline{H}_2$-Al), 1706 (w, Fe-µ$\underline{H}_2$-Al)

EA calc for $C_{69}H_{119}Al_2Fe_2N_4P_6$ (2b.toluene): C 60.48 %, H 8.75 %, N 5.11 %. Found C 59.54 %, H 8.54 %, N 5.00 %

Selected NMR resonances for **2b-[^{13}C]**:

^{1}H NMR (500 MHz, C_6D_6, 298 K): δ 0.56 (d, $^1J_{H-C}$ = 127.4 Hz, 1H, ^{13}C$\underline{H}$CN).

^{13}C NMR (126 MHz, C_6D_6, 298 K): δ 22.13 (br s, 1C, FWHM = 51 Hz, $^{13}\underline{C}$HCN). The ^{13}CH$\underline{C}$N was not observed in ^{13}C{^{1}H}, ^{1}H-^{13}C HMBC, ^{1}H-^{13}C H2BC, ^{1}H-^{13}C ADEQUATE and ^{13}C-^{13}C INADEQUATE experiments. The ^{13}CHC$\underline{N}$ was not observed in ^{14}N and ^{1}H-^{14}N HMBC experiments.

In Situ Preparation of **3a**

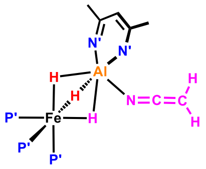

Method for NMR spectroscopic characterisation: In a glovebox, a J. Youngs NMR tube was charged with a solution of **1a** (5 mg, 0.0077 mmol, 1 equiv.) in C_6D_6 (0.5 mL) and MeCN (3.75 µL, 0.0385 mmol, 5 equiv.) was added. The NMR tube was inverted 10 times, and an immediate colour change to light brown-yellow was observed. Due to its thermal instability, **3a** could not be isolated, but the sample was immediately characterised by *in situ* NMR spectroscopy (> 95 % NMR yield).

Method for IR spectroscopic characterisation: In a glovebox, a J. Youngs ampoule was charged with a solution of **1b** (15 mg, 0.0231 mmol, 1 equiv.) in pentane (2 mL) and MeCN (20 µL, 0.383 mmol, 16.6 equiv.) was added resulting in an immediate colour change to yellow-brown. The solution was stirred for 5 min. and the volatiles were removed under vacuum for 15 min. The pale yellow-brown residue was immediately analysed by IR spectroscopy.

^{1}**H NMR** (400 MHz, C_6D_6, 298 K): δ 6.82 (s, 2H, Mes C*H*), 6.74 (s, 2H, Mes C*H*), 5.22 (s, 1H, BDI C*H*), 2.73 (s, 6H, Mes CH_3), 2.27 (s, 6H, Mes CH_3), 2.18 (s, 6H, Mes CH_3), 1.80 (s, 2H, NCCH_2), 1.50 (s, 6H, BDI CH_3), 0.88 (s, 27H, P(CH_3)$_3$), -16.0 (s, 3H, Fe-µH_3-Al).

^{31}**P{^{1}H} NMR** (162 MHz, C_6D_6, 298 K): δ 28.8 (s, 3P).

Selected ^{13}C resonances (from DEPT-135 NMR, 101 MHz, C_6D_6, 298 K): δ 130.0 (Mes *C*H), 128.9 (Mes *C*H), 99.5 (BDI *C*H), 25.9 (m, 9C, P(*C*H_3)$_3$), 23.6 (BDI *C*H_3), 20.6 (Mes *C*H_3), 20.0 (Mes *C*H_3), 19.60 (Mes *C*H_3), 3.8 (NC*C*H_2).

IR (ATR, solid, ν/cm^{-1}): 2099 (m, C=N), 1757 (w, Fe-µH_2-Al), 1706(w, Fe-µH_2-Al)

2.5 In Situ Preparation of 3b

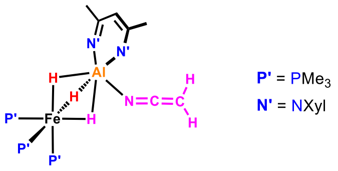

Method for NMR spectroscopic characterisation: In a glovebox, a J. Youngs NMR tube was charged with a solution of **1b** (5 mg, 0.0081 mmol, 1 equiv.) in C_6D_6 and a capillary containing PPh_3 in C_6D_6 was added. MeCN (3.75 µL, 0.072 mmol, 9 equiv.) was added, the NMR tube was quickly removed from the glovebox and the contents frozen in liquid nitrogen. Once thawed, the NMR tube was inverted 10 times, and a colour change to light brown-yellow was observed. The reaction was immediately monitored by NMR spectroscopy, which showed 94 % conversion to **3b**. To get higher resolution $^{13}C\{^1H\}$ data, we repeated the procedure using toluene-[D_8] as the solvent and recorded NMR data at -40 °C (233 K), where **3b** appears to be stable.

3b-[^{13}C] was synthesised using **1b** (5 mg, 0.0081 mmol, 1 equiv.) and MeCN-[2-^{13}C] (3.75 µL, 0.072 mmol, 9 equiv.) using an analogous procedure, and characterised by NMR immediately.

Method for IR spectroscopic characterisation: In a glovebox, a J. Youngs ampoule was charged with a solution of **1b** (15 mg, 0.0231 mmol, 1 equiv.) in pentane (2 mL) and MeCN (20 µL, 0.383 mmol, 16.6 equiv.) was added resulting in an immediate colour change to yellow-brown. The solution was stirred for 5 min. and the volatiles were removed under vacuum for 15 min. The pale yellow-brown residue was immediately analysed by IR spectroscopy.

^{1}H NMR (400 MHz, C6D6): δ 6.99 – 6.89 (m, 6H, Xyl C*H*), 5.20 (s, 1H, BDI C*H*), 2.75 (s, 6H, Xyl C*H*$_3$), 2.27 (s, 6H, Xyl C*H*$_3$), 1.81 (s, 2H, NCC*H*$_2$), 1.46 (s, 6H, BDI C*H*$_3$), 0.86 (br q, J = 4.1, 2.4 Hz, 27H), -16.00 (br s, 3H, Fe-µ*H*$_3$-Al).

DEPT-135 NMR (101 MHz, C_6D_6, 298 K): δ 129.2 (2C, Xyl *C*H), 127.9 (2C, Xyl *C*H), 125.1 (2C, Xyl *C*H), 99.4 (2C, BDI *C*H), 25.7 (m, 9C, P(*C*H$_3$)$_3$), 23.4 (2C, *C*CH$_3$), 19.9 (2C, *C*CH$_3$), 19.5 (2C, *C*CH$_3$), 3.8 (NC*C*H$_2$).

$^{31}P\{^1H\}$ NMR (162 MHz, C_6D_6, 298 K): δ 28.8 (s, 3P).

^{1}H NMR (400 MHz, toluene-[D_8], 298 K): δ 6.91 (s, 2H, Xyl *p*-C*H*), 6.89 (s, 2H, Xyl *m*-C*H*$_3$), 5.13 (s, 1H, BDI C*H*), 2.69 (s, 6H, Xyl C*H*$_3$), 2.25 (s, 6H, Xyl C*H*$_3$), 1.68 (s, 2H, NCC*H*$_2$), 1.42 (s, 6H, BDI C*H*$_3$), 0.85 – 0.81 (m, 27H, P(C*H*$_3$)$_3$), -16.03 (s, 3H, Fe-µ*H*$_3$-Al).

$^{31}P\{^1H\}$ NMR (162 MHz, C_6D_6, 298 K): δ 28.9 (s, 3P).

^{1}H NMR (400 MHz, toluene-[D_8], 233 K): δ 6.91 (t, J = 7.4 Hz, 1H, Xyl *p*-C*H*), 6.86 (dd, J = 7.4, 1.9 Hz, 2H, Xyl *m*-C*H*$_3$), 5.08 (s, 1H, BDI C*H*), 2.73 (s, 6H, Xyl C*H*$_3$), 2.24 (s, 6H, Xyl C*H*$_3$), 1.82 (s, 2H, NCC*H*$_2$), 1.38 (s, 6H, BDI C*H*$_3$), 0.81 – 0.71 (m, 27H, P(C*H*$_3$)$_3$), -15.96 (s, 1H, Fe-µ*H*$_3$-Al).

$^{13}C\{^1H\}$ NMR (101 MHz, toluene-[D_8], 233 K): δ 167.6 (2C, BDI *C*N), 158.6 (2C, Xyl *C*N), 148.7 (2C, Xyl *C*CH$_3$), 137.1 (2C, Xyl *C*CH$_3$), 133.9(2C, Xyl *C*H, 130.0 (2C, Xyl *C*H), 128.9 (2C, Xyl *C*H), 126.0 (Xyl *C*H), 100.2 (BDI *C*H), 26.2 (m, 9C, P(*C*H$_3$)$_3$), 24.5 (2C, *C*CH$_3$), 23.5 (2C, *C*CH$_3$), 5.3 (NC*C*H$_2$). The 3rd *C*CH$_3$ was not detected as it is likely overlapped by the solvent residual peak.

$^{31}P\{^1H\}$ NMR (162 MHz toluene-[D_8], 233 K) δ 30.0 (s, 3P).

IR (ATR, solid, ν/cm^{-1}): 2084 (m, C=N), 1756 (w, Fe-µ*H*$_2$-Al), 1717 (w, Fe-µ*H*$_2$-Al)

Selected resonances for **3b-[^{13}C]**:

^{1}H NMR (400 MHz, C_6D_6): 1.81 (d, 2H, $^1J_{C\text{-}H}$ = 166.6 Hz, NC***C***H_2).

DEPT-135 NMR (101 MHz, C_6D_6, 298 K): δ 3.75 (NC*C*H_2).

3 NMR monitoring studies

3.1 NMR monitoring of the reaction of **1a** with 1.0 equiv. of CH_3CN

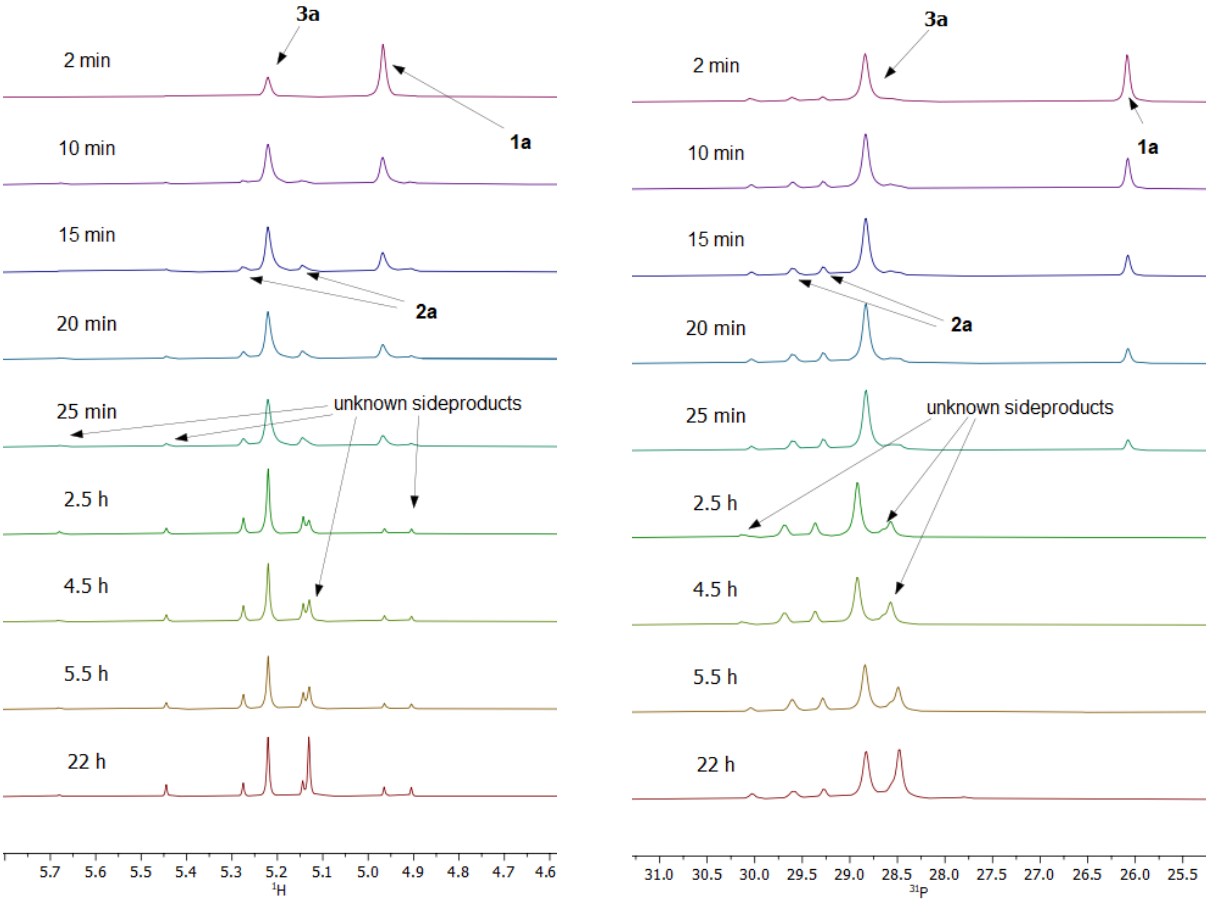

Figure S 1 NMR monitoring of the reaction of **1a** with 1.0 equiv. of CH_3CN. Left: stacked ^{1}H NMRs (BDI backbone region only), Right: stacked $^{31}P\{^{1}H\}$ NMRs (relevant region only).

3.2 NMR monitoring of the reaction of **1b** with 0.5 equiv. of CH_3CN

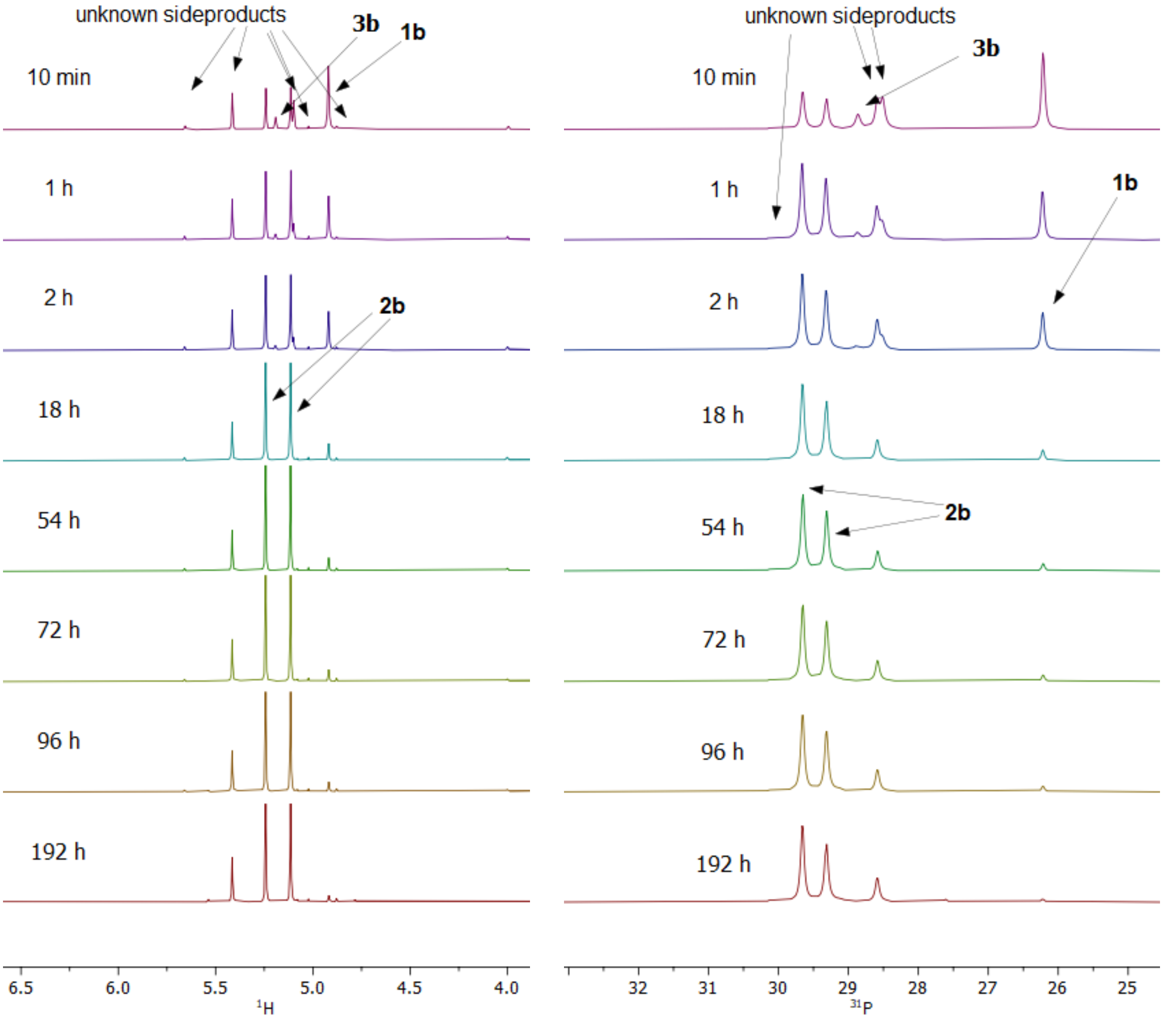

Figure S 2 NMR monitoring of the reaction of **1b** with 0.5 equiv. of CH_3CN. Left: stacked ^1H NMRs (BDI backbone region only), Right: stacked $^{31}P\{^1H\}$ NMRs (relevant region only)

4 X-Ray data

4.1 Crystal data table

compound	2a	2b
**CCDC No.**	2224389	2224390
**formula**	$C_{66}H_{119}Al_2Fe_2N_5P_6$	$C_{62}H_{111}Al_2Fe_2N_5P_6$
**solvent**	C_6H_6	C_7H_8
**formula weight**	1412.24	1370.16
**colour, habit**	orange tablet	pale orange plate
**temperature / K**	173	173
**crystal system**	triclinic	monoclinic
**space group**	*P*–1 (no. 2)	$P2_1/c$ (no. 14)
***a* / Å**	11.8578(3)	22.6316(6)
***b* / Å**	13.2859(4)	11.7705(2)
***c* / Å**	14.2902(4)	29.1563(7)
**α / deg**	105.009(2)	90
**β / deg**	91.866(2)	93.302(2)
**γ / deg**	113.041(2)	90
**V / Å^3**	1978.18(9)	7753.9(3)
Z	1	4
**D_c / g cm^{-3}**	1.185	1.174
**radiation used**	Mo-Kα	Cu-Kα
**μ / mm^{-1}**	0.551	4.687
**no. of unique reflections**		
**measured (R_{int})**	9070 (0.0411)	14854 (0.0744)
**obs, $\|Fo\|>4\sigma(\|Fo\|)$**	7267	8470
**completeness (%)[a]**	99.9	98.5
**no. of variables**	435	893
**R_1 (obs), wR_2 (all)[b]**	0.0392, 0.1036	0.0601, 0.1624

Table S 1A summary of the crystallographic data for the structures of **2a** and **2b**. Data were collected using Agilent Xcalibur 3 E (**2a**) and Xcalibur PX Ultra A (**2b**) diffractometers, and the structures were solved and refined using the OLEX2,[3] SHELXTL[4] and SHELX-2013[5,6] program systems. [a] Completeness to 0.84 Å resolution. [b] $R_1 = \Sigma||F_o| - |F_c||/\Sigma|F_o|$; $wR_2 = \{\Sigma[w(F_o^2 - F_c^2)^2] / \Sigma[w(F_o^2)^2]\}^{1/2}$; $w^{-1} = \sigma_2(F_o^2) + (aP)^2 + bP$.

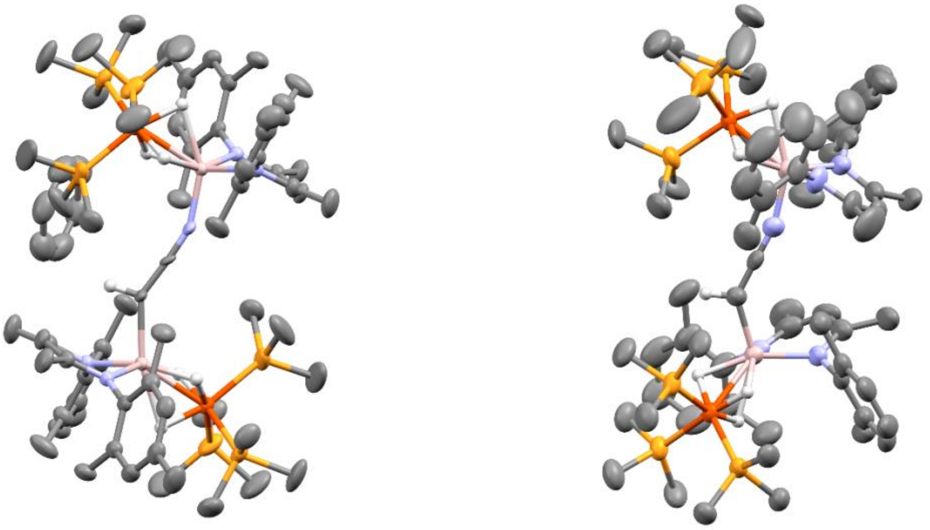

2a **2b**

Figure S 3 Left: The crystal structure of **2a** (50% probability ellipsoids). The structure sits across a centre of symmetry with the bridging $[CHCN]^{2-}$ unit being disordered about the centre of symmetry. Most hydrogens, the second orientation of the bridging $[CHCN]^{2-}$ unit, and the included solvent molecule removed from clarity. Right: The crystal structure of **2b** (50% probability ellipsoids). Most hydrogens, the minor occupancy orientation of disordered atoms, and the included solvent molecule removed from clarity.

4.2 The X-ray structure of 2a

The structure of **2a** was found to sit across a centre of symmetry situated near to C41 (*ca*. 0.21 Å away). However, due to the asymmetric nature of the N40-based $[CHCN]^{2-}$ bridging unit, the actual molecule cannot possess a centre of symmetry, making the bridging unit inherently disordered (the rest of the complex can "obey" the inversion centre). This was handled by modelling one 50% occupancy $[CHCN]^{2-}$ unit, with a second orientation of the same occupancy being generated by operation of the centre of symmetry. No restraints were applied, and the three unique non-hydrogen atoms were refined anisotropically. The C42–H hydrogen atom could be located from a Δ*F* map, but was added in an idealised position and allowed to ride on its parent atom. The three unique Al– μ-H–Fe bridging hydrogen atoms were all located from Δ*F* maps and refined freely.

4.3 The X-ray crystal structure of 2b

The P6-based trimethylphosphine was found to be rotationally disordered between two positions at a ratio of ca. 0.79:0.21. Only the non-hydrogen atoms of the major component were modelled anisotropically, the rest isotropically, with P6 being common to both. The N5-based xylyl group was found to disordered between two positions at a ratio of ca. 0.76:0.24. Only the non-hydrogen atoms of the major component were modelled anisotropically, the rest isotropically, with N5 being common to both. The asymmetric unit was found to contain one molecule of toluene that is disordered between two positions at a ratio of ca. 0.66:0.34. Again, only the non-hydrogen atoms of the major component were modelled anisotropically, the rest isotropically. The C2-H hydrogen atom could be found in the Δ*F* map, but was added in an idealised position and allowed to ride on its parent atom. All six crystallographically independent Al–μ-H–Fe hydrogens atoms were found in the Δ*F* maps and freely refined.

5 Computational methods

5.1 General considerations

DFT calculations were run using Gaussian 09 (Revision D.01).[7] NBO analysis was performed using the NBO v6.0 version program.[8] QTAIM analysis was conducted with the AIMAll package.[9]

For optimisations Al and Fe centres were described with Stuttgart SDDAll ECP and associated basis sets, and the 6-31G** basis sets were used for all other atoms. Single point basis set corrections were applied using the def2-TZVPP basis set for all atoms. Geometry optimisation calculations were performed without symmetry constraints. Frequency analyses for all stationary points were performed using the enhanced criteria to confirm the nature of the structures as either minima (no imaginary frequency) or transition states (only one imaginary frequency). Solvent corrections were applied using the polarizable continuum model (PCM). Dispersion corrections were applied using Grimme's D3 correction.

Ground state structures were optimised at a ωB97XD // 6-31G** / SSDAll level of theory. These structures were used as input for NBO, QTAIM and ETS-NOCV calculations. Free energy profiles were calculated at a B3PW91 // 6-31G** / SSDAll [optimisation]; B3PW91 // def2-TZVPP [single point] level of theory including solvent and dispersion corrections directly in the optimisations. For structural/electronic studies along the reaction coordinate the structures optimised with the B3PW91 functional were used as inputs with the ωB97XD functional. All calculation were carried out on the singlet energy surface.

ETS-NOCV calculations were performed in the Orca 4.2.1 quantum chemistry software package[10,11] with optimised geometries obtained at a ωB97x // 6-31G** / SSDAll level of theory. The calculations were run using the ωB97x functional with the def2-tzvpp basis set. Calculations were performed with the resolution of identity approximation for the Coulomb integrals, and chain of spheres approximation for the exchange integrals (RIJCOSX) with the def2/j auxiliary basis set.[12]

Computed structure visualisation were created using CYLView version 1.0.600 BETA,[13] and visualisations of isosurfaces using VMD.[14]

5.2 Alternative pathway for the double deprotonation of CH_3CN by **1b**

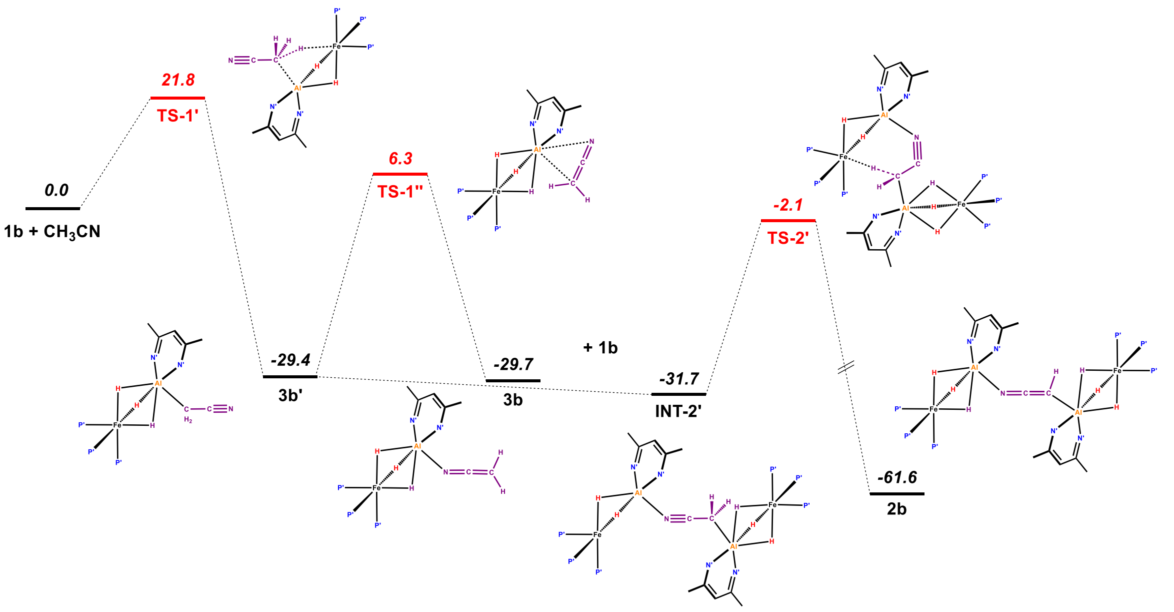

Figure S 4 Alternative pathway for the formation of **2b**.

5.3 Computed geometries along the reaction coordinates using **1b**

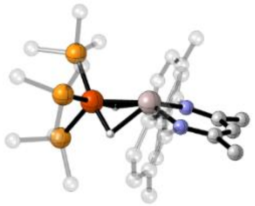

1b

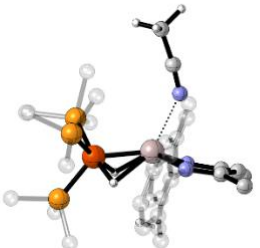

INT-1b

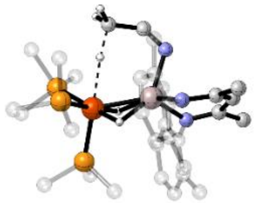

TS-1b

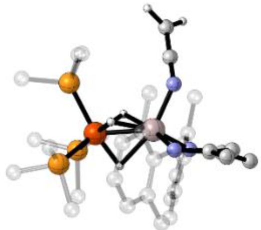

3b

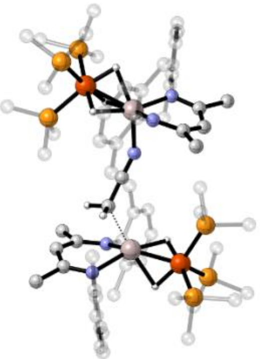

INT-2b

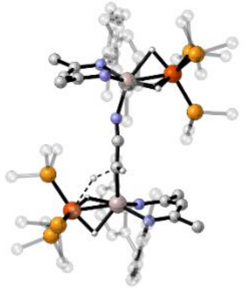

TS-2b

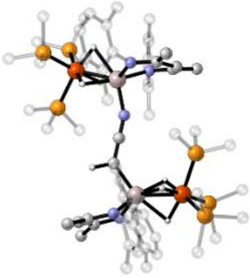

2b

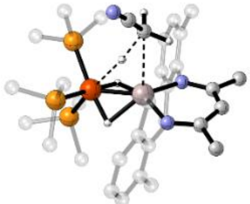

TS-1b’

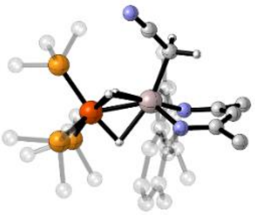

3b’

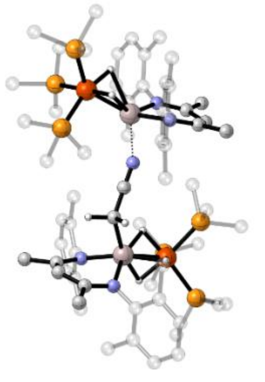

INT-2b’

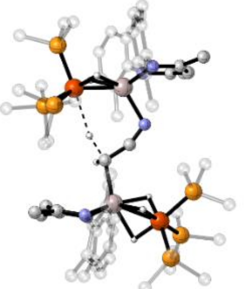

TS-2b’

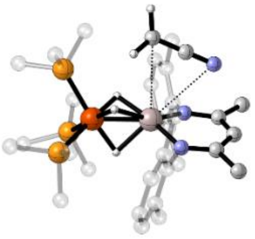

TS-1b’’

5.4 Functional and basis set testing for single point corrections

The potential energy surfaces for both pathways for the double deprotonation of CH_3CN were calculated using both **1a** and **1b**, producing very similar geometries and slightly different energies for analogous species.

Species	Mes (**a**) [a]	[ab]	[ac]	[ad]	Xyl (**b**) [a]	[ab]	[ac]	[ad]
**INT-1**	7.0	**7.0**	6.2	8.2	6.5	**6.5**	6.1	7.5
**TS-1**	17.3	**14.2**	13.6	22.0	17.1	**14.0**	14.2	21.9
**3**	-22.7	**-29.4**	-20.8	-24.6	-23.0	**-29.7**	-19.9	-24.9
**INT-2**	-23.4	**-26.6**	-26.2	-25.4	-24.2	**-27.4**	-25.9	-25.5
**TS-2**	-10.1	**-18.1**	-11.6	-7.7	-11.1	**-19.4**	-11.8	-8.0
**2**	-53.1	**-60.3**	-50.9	-55.9	-54.2	**-61.6**	-49.1	-55.5
**TS-1'**	26.9	**22.1**	24.5	30.0	26.5	**21.8**	25.1	30.3
**3'**	-26.2	**-29.0**	-24.4	-29.7	-26.5	**-29.4**	-23.9	-29.8
**INT-2'**	-30.6	**-31.4**	-30.1	-33.2	-31.0	**-31.7**	-33.0	-32.2
**TS-2'**	2.7	**-1.3**	1.6	7.0	1.9	**-2.1**	3.9	7.1
**TS-1''**	13.0	**6.6**	12.0	9.3	12.7	**6.3**	12.4	10.6

Table S 2 Energies of the stationary points along the reaction coordinate, Gibbs free energies in kcal/mol). [a] B3PW91 // 6-31G** / SDDAll / GD3 / PCM (benzene) [optimisation] [b] B3PW91 // def2-TZVPP / GD3 / PCM (benzene) [single point] [c] M06L // 6-31G** / SDDAll / GD3 / PCM (benzene) [single point] [d] ωB97XD // 6-31G** / SDDAll / PCM (benzene) [single point]

5.5 Structural computations

NBO calculations were performed on all species along the reaction coordinate for both Mes (**a**) and Xyl (**b**). The tables below also contain data for **2a-b** optimised with the ωB97XD functional. [a] B3PW91 // 6-31G** / SDDAll / GD3 / PCM (benzene) [optimisation] [e] ωB97XD // 6-31G** / SDDAll [optimisation] [f] ωB97XD // 6-31G** / SDDAll / PCM (benzene) [NBO] [g] ωB97XD // 6-31G** / SDDAll [NBO].

5.5.1 NBO (v6.0)[8] data along the reaction pathways

R = Mes (**a**)

	**1**^{af}	CH_3CN^{af}	**INT-1**^{af}	**TS-1**^{af}	**3**^{af}	**INT-2**^{af}	**TS-2**^{af}	**2**^{af}	**2**^{eg}	**TS-1'**^{af}	**3'**^{af}
**MBI**											
Fe-Al	1.10		0.95	0.64	0.53	0.55	0.52	0.52	0.51		
Fe-µH	0.79		0.82	0.71	0.71	0.71	0.70	0.73	0.73		
	0.69		0.78	0.65	0.75	0.73	0.74	0.74	0.73		
					0.75	0.73	0.77	0.81	0.82		
[(Fe)----H]				0.41							
Al-µH	0.13		0.09	0.18	0.23	0.23	0.23	0.23	0.24		
	0.23		0.13	0.28	0.21	0.24	0.23	0.23	0.23		
					0.20	0.20	0.17	0.12	0.12		
[(Al)----H]				0.07							
Al-N			0.24	0.56	0.53	0.33	0.40	0.35	0.40		
N-C		2.85	2.73	2.29	2.11	2.51	2.28	2.19	2.19	2.82	2.80
C-C		0.99	1.02	1.24	1.69	1.34	1.47	1.76	1.75	1.18	1.07
C-H		0.93	0.92	0.94	0.93	0.88	0.89	0.87	0.87	0.92	0.91
		0.93	0.93	0.94	0.93	0.89				0.93	0.91
		0.93	0.93								
[(C)----H]				0.47			0.56			0.30	
Al-C						0.38	0.49	0.73	0.74	0.06	0.59
Fe-Al						0.93	0.56	0.47	0.47	0.71	0.53
Fe-µH						0.80	0.74	0.75	0.75	0.73	0.72
						0.77	0.60	0.77	0.75	0.65	0.75
								0.80	0.81		0.76
[(Fe)----H]							0.30			0.26	
Al-µH						0.12	0.17	0.19	0.19	0.20	0.20
						0.15	0.29	0.20	0.21	0.26	0.21
								0.13	0.12		0.17
[(Al)----H]							0.05			0.38	
**NPA charge**											
Fe	-1.00		-1.12	-0.90	-0.85	-0.84	-0.85	-0.87	-0.82		
Al(N)	1.24		1.41	1.58	1.73	1.72	1.73	1.75	1.75		
µH	-0.15		-0.15	-0.19	-0.18	-0.18	-0.18	-0.17	-0.18		
	-0.19		-0.17	-0.2	-0.17	-0.18	-0.18	-0.17	-0.18		
						-0.18	-0.18	-0.17	-0.17		
N		-0.33	-0.42	-0.72	-0.83	-0.70	-0.79	-0.93	-0.93	-0.45	-0.48
(N)C		0.28	0.41	0.27	0.41	0.51	0.48	0.43	0.43	0.27	0.35
C		-0.81	-0.82	-0.81	-0.84	-1.07	-1.2	-1.18	-1.18	-0.94	-1.17
(C)H		0.29	0.30	0.27	0.25	0.29	0.28	0.26	0.26	0.24	0.28
		0.29	0.30	0.26	0.25	0.28				0.25	0.28
		0.29	0.30								
[(C)----H]				0.13			0.25			-0.01	
Fe						-1.11	-1.02	-0.89	-0.84	-0.91	-0.85
Al(C)						1.45	1.68	1.70	1.69	1.61	1.73
µH						-0.16	-0.2	-0.17	-0.17	-0.17	-0.19
						-0.18	-0.21	-0.16	-0.17	-0.19	-0.17
								-0.16	-0.16		-0.18

	R = Mes (a)										
	**1**[af]	CH_3CN[af]	**INT-1**[af]	**TS-1**[af]	**3**[af]	**INT-2**[af]	**TS-2**[af]	**2**[af]	**2**[eg]	**TS-1'**[af]	**3'**[af]
**WBI**											
Fe-Al	0.53		0.44	0.24	0.16	0.16	0.16	0.15	0.15		
Fe-µH	0.42		0.44	0.42	0.42	0.42	0.43	0.4	0.43		
	0.44		0.43	0.41	0.43	0.42	0.42	0.42	0.43		
					0.43	0.43	0.42	0.412	0.45		
[(Fe)----H]				0.20							
Al-µH	0.25		0.20	0.26	0.27	0.27	0.25	0.26	0.26		
	0.21		0.22	0.28	0.26	0.27	0.26	0.26	0.27		
					0.25	0.26	0.26	0.22	0.22		
[(Al)----H]				0.12							
Al-N			0.17	0.33	0.34	0.30	0.32	0.35	0.35		
N-C		2.91	2.79	2.36	2.23	2.45	2.23	2.10	2.10	2.74	2.78
C-C		1.08	1.08	1.31	1.64	1.33	1.40	1.70	1.71	1.20	1.16
C-H		0.89	0.88	0.90	0.90	0.86	0.86	0.86	0.86	0.90	0.88
		0.89	0.88	0.90	0.90	0.86				0.90	0.87
		0.89	0.88							0.29	
[(C)----H]				0.47			0.53				
Al-C						0.19	0.28	0.44	0.45	0.05	0.33
Fe-Al						0.42	0.20	0.15	0.15	0.25	0.16
Fe-µH						0.43	0.40	0.42	0.43	0.42	0.42
						0.42	0.40	0.43	0.43	0.40	0.43
								0.44	0.45	0.23	0.43
[(Fe)----H]							0.14			0.23	
Al-µH						0.24	0.27	0.26	0.26	0.25	0.27
						0.21	0.34	0.25	0.26	0.29	0.26
								0.22	0.22		0.25
[(Al)----H]							0.09			0.32	

						R = Xyl (**b**)					
	**1**[af]	CH_3CN[af]	**INT-1**[af]	**TS-1**[af]	**3**[af]	**INT-2**[af]	**TS-2**[af]	**2**[af]	**2**[eg]	**TS-1'**[af]	**3'**[af]
**MBI**											
Fe-Al	1.10		0.95	0.64	0.53	0.55	0.52	0.52	0.51		
Fe-μH	0.79		0.82	0.72	0.70	0.71	0.69	0.74	0.73		
	0.69		0.78	0.65	0.75	0.72	0.73	0.74	0.73		
					0.75	0.74	0.76	0.81	0.82		
[(Fe)----H]				0.41							
Al-μH	0.12		0.09	0.18	0.24	0.23	0.22	0.23	0.24		
	0.23		0.13	0.28	0.32	0.24	0.23	0.23	0.24		
					0.20	0.20	0.17	0.12	0.12		
[(Al)----H]				0.07							
Al-N			0.24	0.56	0.53	0.33	0.43	0.39	0.40		
N-C		2.85	2.73	2.29	2.11	2.51	2.19	2.19	2.19	2.82	2.80
C-C		0.99	1.02	1.24	1.69	1.34	1.53	1.76	1.75	1.18	1.07
C-H		0.93	0.92	0.93	0.93	0.89	0.88	0.87	0.87	0.92	0.91
		0.93	0.93	0.93	0.93	0.89				0.93	0.91
		0.93	0.93								
[(C)----H]				0.47			0.50			0.30	
Al-C						0.38	0.51	0.73	0.74	0.06	0.60
Fe-Al						0.95	0.53	0.47	0.47	0.71	0.53
Fe-μH						0.80	0.60	0.77	0.75	0.73	0.72
						0.77	0.73	0.76	0.75	0.65	0.75
								0.80	0.81		0.76
[(Fe)----H]							0.35			0.26	
Al-μH						0.12	0.29	0.20	0.19	0.20	0.21
						0.15	0.17	0.19	0.21	0.37	0.21
								0.13	0.12		0.17
[(Al)----H]							0.06			0.38	
**NPA charge**											
Fe	-1.00		-1.12	-0.90	-0.85	-0.84	0.94	-0.87	-0.82		
Al(N)	1.24		1.41	1.58	1.73	1.74	1.70	1.75	1.74		
μH	-0.15		-0.15	-0.19	-0.17	-0.18	-0.16	-0.17	-0.18		
	-0.20		-0.17	-0.20	-0.18	-0.18	-0.16	-0.17	-0.18		
					-0.18	-0.18	-0.15	-0.17	-0.17		
N		-0.33	-0.42	-0.72	-0.83	-0.7	-0.80	-0.93	-0.93	-0.45	-0.48
(N)C		0.28	0.41	0.37	0.41	0.51	0.42	0.43	0.44	0.27	0.35
C		-0.81	-0.82	-0.81	-0.84	-1.07	-1.17	-1.19	-1.18	-0.94	-1.17
(C)H		0.29	0.30	0.27	0.25	0.27	0.28	0.26	0.26	0.24	0.27
		0.29	0.30	0.26	0.25	0.28				0.25	0.28
		0.29	0.30								
[(C)----H]				0.13			0.20			-0.01	
Fe						-1.11	-1.03	-0.89	-0.84	-0.90	-0.85
Al(C)						1.45	1.67	1.70	1.69	1.61	1.73
μH						-0.16	-0.18	-0.17	-0.17	-0.18	-0.19
						-0.19	-0.19	-0.16	-0.17	-0.20	-0.17
								-0.16	-0.16		-0.18

	R = Xyl (**b**)										
	**1**[af]	CH_3CN[af]	**INT-1**[af]	**TS-1**[af]	**3**[af]	**INT-2**[af]	**TS-2**[af]	**2**[af]	**2**[eg]	**TS-1'**[af]	**3'**[af]
**WBI**											
Fe-Al	0.53		0.44	0.24	0.16	0.16	0.16	0.15	0.15		
Fe-µH	0.42		0.44	0.42	0.43	0.43	0.42	0.42	0.43		
	0.44		0.43	0.41	0.43	0.42	0.41	0.42	0.43		
					0.42	0.42	0.41	0.44	0.45		
[(Fe)----H]				0.20							
Al-µH	0.25		0.22	0.26	0.26	0.27	0.25	0.26	0.27		
	0.21		0.20	0.27	0.27	0.27	0.25	0.26	0.27		
					0.25	0.26	0.25	0.22	0.22		
[(Al)----H]				0.12							
Al-N			0.17	0.33	0.34	0.30	0.34	0.35	0.35		
N-C		2.91	2.79	2.36	2.23	2.45	2.23	2.10	2.10	2.74	2.79
C-C		1.08	1.08	1.31	1.64	1.34	1.44	1.70	1.71	1.21	1.16
C-H		0.89	0.88	0.90	0.90	0.86	0.86	0.86	0.86	0.90	0.88
		0.89	0.88	0.90	0.90	0.86				0.90	0.87
		0.89	0.88								
[(C)----H]				0.47			0.86			0.28	
Al-C						0.19	0.30	0.44	0.44	0.05	0.33
Fe-Al						0.42	0.18	0.15	0.15	0.25	0.16
Fe-µH						0.43	0.39	0.42	0.43	0.42	0.42
						0.42	0.36	0.43	0.43	0.40	0.43
								0.44	0.45		0.43
[(Fe)----H]							0.17			0.23	
Al-µH						0.21	0.26	0.26	0.26	0.25	0.27
						0.24	0.34	0.25	0.26	0.29	0.26
								0.22	0.22		0.25
[(Al)----H]							0.10			0.32	

Table S 3 NBO data along the reaction coordinate for top: R = Mes (**a**) and bottom: R = Xyl (**b**). The table includes MBIs (Mayer Bond Indices), WBIs (Wiberg Bond Indices) and NPA (Natural Population Analysis) charges.

QTAIM[9] data for **2a-b**

	**2a**[e]			**2b**[e]		
	**ρ(r)**	**∇2 (r)**	**ellipticity**	**ρ(r)**	**∇2 (r)**	**ellipticity**
Fe-Al	0.0497	0.0863	7.3806	0.0498	0.0867	6.9308
Fe-μH	0.1050	0.2578	0.1367	0.1074	0.2582	0.1381
	0.1040	0.2555	0.1312	0.1038	0.2560	0.1328
	0.1054	0.2202	0.0626	0.1051	0.2208	0.0641
Al-N	0.0640	0.4740	0.0358	0.0640	0.4738	0.0362
N-C	0.4182	-0.3130	0.0409	0.4183	-0.3132	0.0302
C-C	0.3200	-0.6419	0.4163	0.3199	-0.6418	0.4174
C-H	0.2605	-0.8131	0.0229	0.2605	-0.8131	0.0323
Al-C	0.0676	0.3219	0.0670	0.0680	0.3220	0.0675
Fe-Al	0.0481	0.0839	2.9848	0.0482	0.0842	2.9461
Fe-μH	0.1048	0.2482	0.1163	0.1047	0.2531	0.1208
	0.1036	0.2506	0.1198	0.1034	0.2488	0.1178
	0.1076	0.2209	0.0609	0.1075	0.2212	0.0616

Table S 4 QTAIM bond data for **2a** and **2b**.

**QTAIM charge**	**2a**[e]	**2b**[e]
Fe	0.39	0.39
Al(N)	2.44	2.44
μH	-0.50	-0.50
	-0.49	-0.50
	-0.44	-0.44
N	-1.79	-1.79
(N)C	0.60	-0.60
C	-0.44	0.44
(C)H	0.00	0.00
Fe	0.38	0.38
Al(C)	2.39	2.39
μH	-0.49	-0.49
	-0.49	-0.49
	-0.44	-0.44

Table S 5 QTAIM atomic charge data for **2a** and **2b**.

5.6 Analysis of the M–[CHCN]–M bonding in **2b**

5.6.1 Available NBOs for the bonding of the bimetallic Fe–Al fragments to the $[CHCN]^{2-}$ dianion in **2b**

NBO (CHK) no.	Description	Isosurface
324 (331)	Empty N–Al s	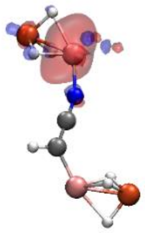
325 (350)	Empty N–Al p_z	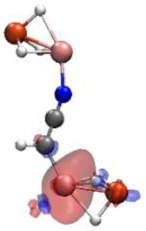
326 (352)	Empty N–Al p_x	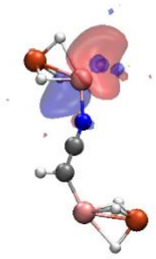
327 (354)	Empty N–Al p_y	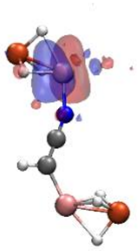
328 (330)	empty C–Al s	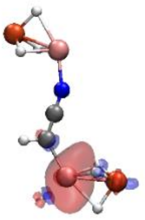

329 (349)	Empty C–Al p_z	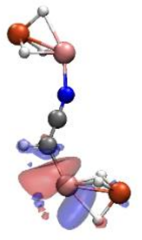
330 (351)	Empty C–Al p_y	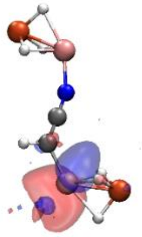
331 (353)	Empty C–Al p_x	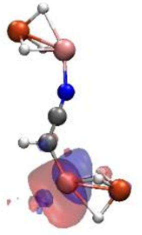
223 (258)	N–C σ	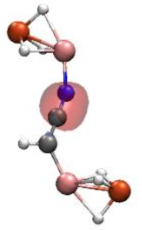
224 (259)	N–C π_x	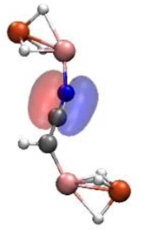

225 (278)	N–C–π_y	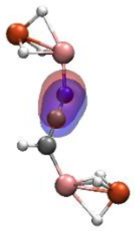
115 (301)	N sp^2_z lone pair	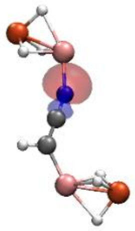
430 (332)	N–C π_y*	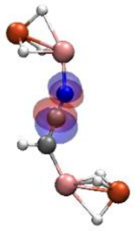
429 (355)	N–C π_x*	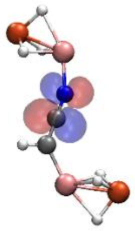
428 (390)	N–C σ*	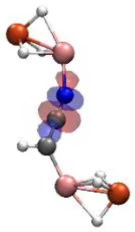

226 (279)	C–C σ	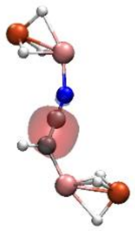
431 (410)	C–C σ*	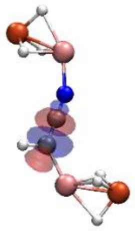
227 (290)	C–H σ	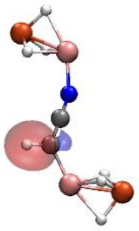
116 (310)	C sp^2_z lone pair	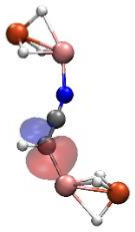
117 (315)	C p_y lone pair	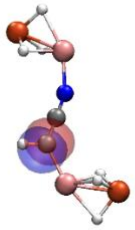

432 (446)	C–H σ*	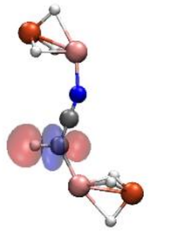
54 (75)	C 1s	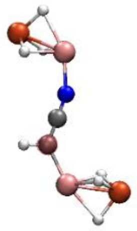

5.6.2 Second order perturbation theory analysis

NBO (v 6.0)[8] places the $[CHCN]^{2-}$ fragment and the two Al nuclei in separate units and thus describes the M–[CHCN]–M bonding via second order perturbations. The main contributors (>10 kcal mol^{-1}) to the second order perturbation theory description of the bonding are the following:

**Bond**	**Donor**		**Acceptor**		**Energy** /kcal mol^{-1}
	NBO (CHK) no.	description	NBO (CHK) no.	description	
N–Al	115 (301)	N sp^2_z	325 (350)	empty N–Al p_z	95.3
	115 (301)	N sp^2_z	324 (331)	empty N–Al s	34.1
	223 (258)	N–C σ	325 (350)	empty N–Al p_z	24.4
	115 (301)	N sp^2_z	326 (352)	empty N–Al p_x	10.5
C–Al	116 (310)	C sp^2_z lone pair	329 (349)	empty C–A p_z	106.9
	116 (310)	C sp^2_z lone pair	328 (330)	empty C–Al s	83.3
	226 (279)	C–C(N) σ	329 (349)	empty C–A p_z	24.9
	117 (315)	C sp^2_y lone pair	330 (351)	empty C–Al p_y	18.9
	227 (290)	C–H σ	329 (349)	empty C–A p_z	18.4
	54 (75)	C 1s	329 (349)	empty C–A p_z	11.2

Table S 6 Dominant second order perturbations for the NBO description of the bonding in the M_2-[CHCN] fragment of **2b**.

5.6.3 ETS-NOCV description

Splitting **2b** at each FeAl---[CHCN] bond allowed us to quantify the bonding, as well as visualise it in terms of the charge flow between the fragments. Splitting **2b** into three fragments ([FeAl]---[CHCN]---[FeAl]) and performing NBO calculations gives a matching bonding description. All isosurfaces are visualised over the same view for easy comparison. For ETS-NOCV calculations: charge flow from blue to red.

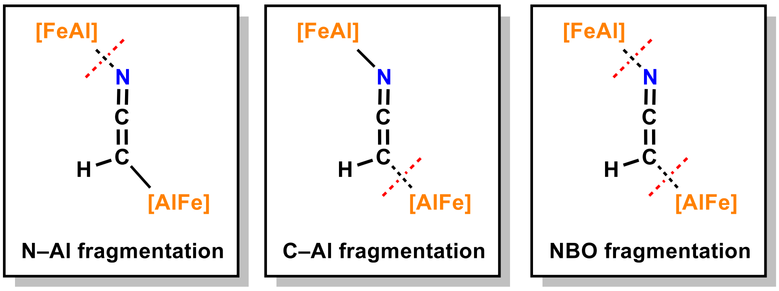

	ETS-NOCV			MO	
**Bond**	**E_{orb}** (kcal mol^{-1})	**Δρ** (kcal mol^{-1})	**Isosurface**	**Donor**	**Acceptor**
N–Al	-181.9	$\Delta\rho_1$ = -26.1 (14.3 %)	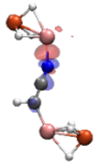	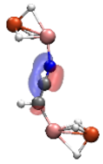	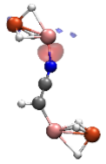
		$\Delta\rho_2$ = -19.2 (10.6 %)	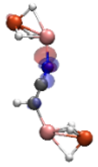	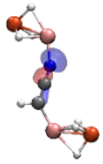	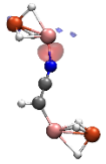
C–Al	-197.9	$\Delta\rho_1$ = -50.0 (25.3 %)	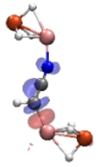	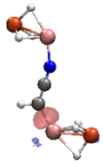	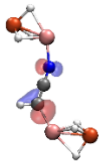

Table S 7 ETS-NOCV and matching fragmented MO description of the bonding in the M_2–[CHCN] fragment of **2b**.

5.7 Spectroscopic simulations

NMR chemicals shifts of the [C̲HnCN] nuclei as well as prediction of the IR vibration for the C=N bonds were performed for **2a-b** and CH_3CN for validation, as well as for **3a-b**, and **3a-b'** to aid assignment of the intermediate.

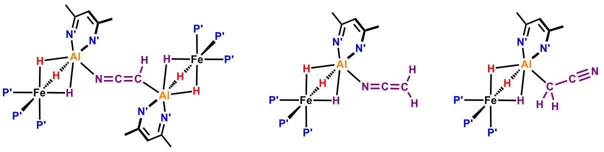

2a (N' = NMes) **2b** (N' = NXyl) **3a** (N' = NMes) **3b** (N' = NXyl) **3a'** (N' = NMes) **3b'** (N' = NXyl)

**Spectroscopic method**	Exp/DFT	R = Mes (**a**)			R = Xyl (**b**)			CH_3CN
		**2**	**3**	**3'**	**2**	**3**	**3'**	
**IR**	Experiment	2029[a]	2099[a]	–	2038[a]	2084[a]	–	2251[b]
	DFT	2033[c]	2076[c]	2216[c]	2032[c]	2076[c]	2216[c]	2278[d]
**NMR**	Experiment	–	3.8[e]	–	22.1[f]	3.8[e]	–	0.2[12]
	DFT	22.5[g]	7.7[g]	-0.9[g]	22.2[g]	7.9[g]	-0.9[g]	0.2[g]

Table S 8 Comparison of experimental and simulated spectroscopic data for **2**, **3**, and **3'**.

[a] ATR IR (solid), 298 K

[b] ATR IR (liquid), 298 K

[c] ωB97XD // 6-31G** / SDDAll, scale factor = 0.948[15]

[d] ωB97XD // 6-31G** / SDDAll / solvent=acetonitrile, scale factor = 0.948[15]

[e] detected by DEPT-135 NMR (C_6D_6, 298 K)

[f] detected by $^{13}C\{^1H\}$ NMR for **2-[^{13}C]** (C_6D_6, 298 K)

[g] B3PW91 // 6-31G** / SDDAll / GD3 / PCM (benzene) [optimisation] // B3PW91 // def2-TZVPP / GD3 / PCM (benzene) / GIAO [NMR]. Referenced to C̲H_3CN (shielding = 199.5, 0.03 ppm).

6 NMR spectra of isolated compounds

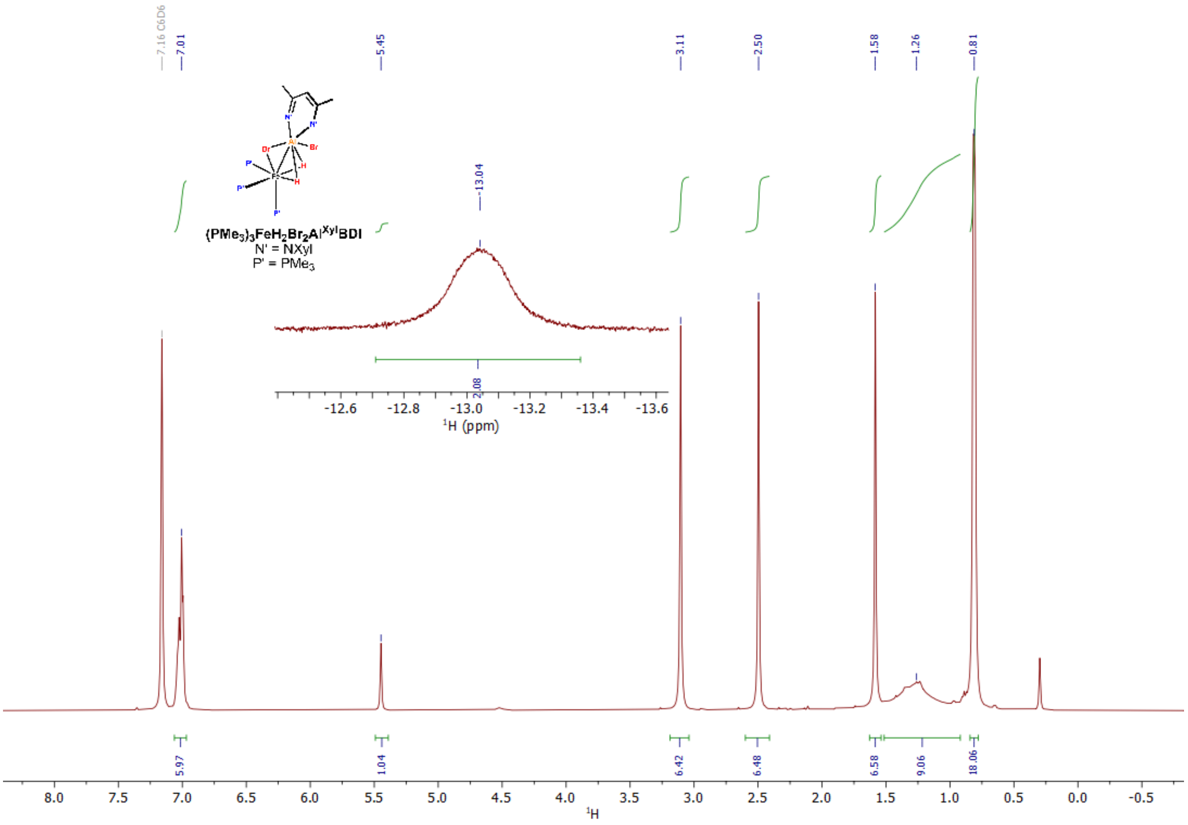

Figure S 5 ^{1}H NMR of $(PMe_3)FeH_2Br_2Al^{Xyl}BDI$ (400 MHz, C_6D_6, 298 K)

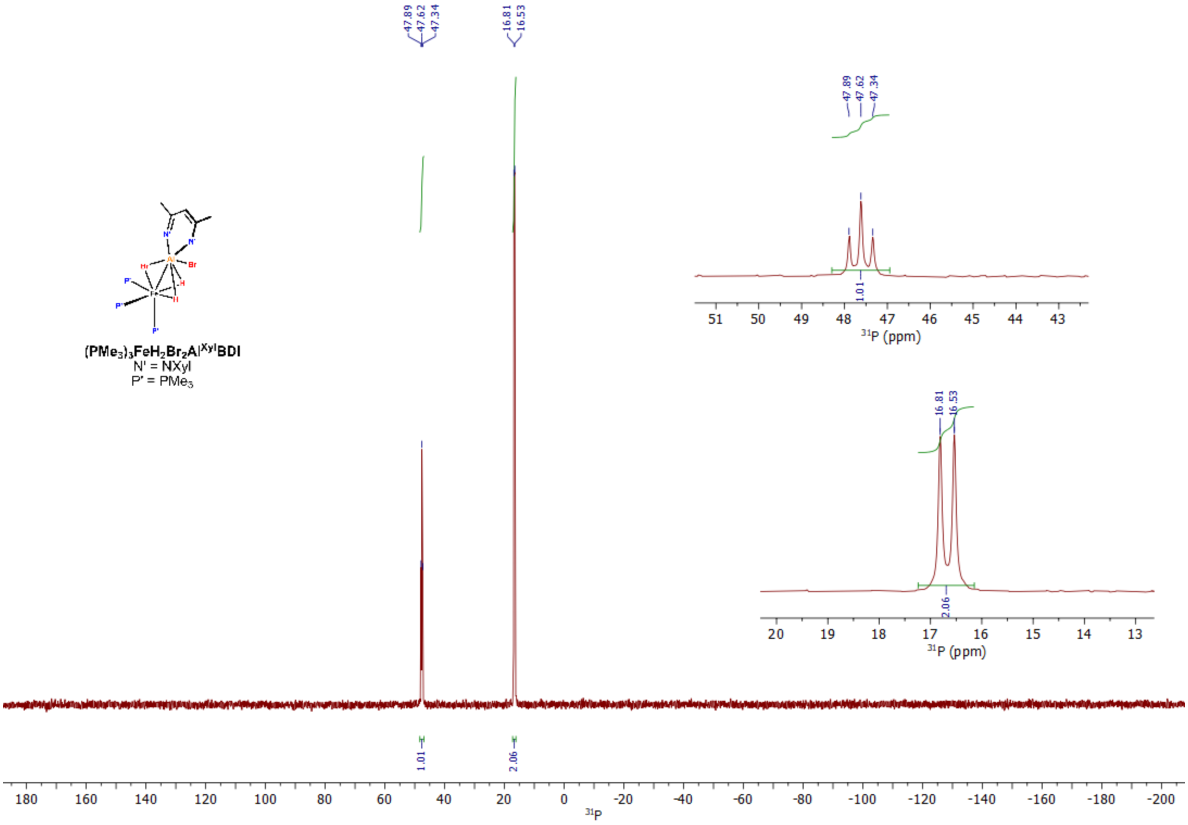

Figure S 6 $^{31}P\{^{1}H\}$ NMR of $(PMe_3)FeH_2Br_2Al^{Xyl}BDI$ (162 MHz, C_6D_6, 298 K)

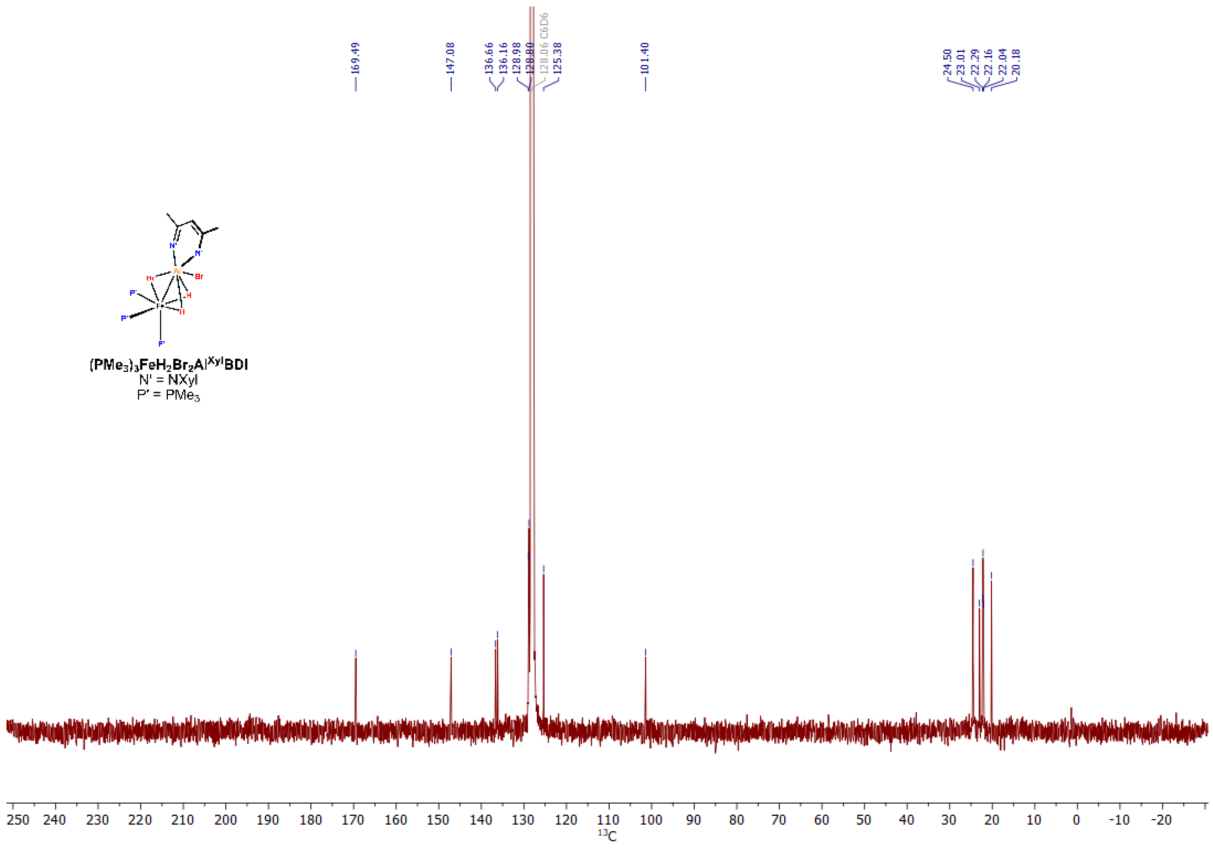

Figure S 7 $^{13}C\{^{1}H\}$ NMR of $(PMe_3)FeH_2Br_2Al^{Xyl}BDI$ (101 MHz, C_6D_6, 298 K).

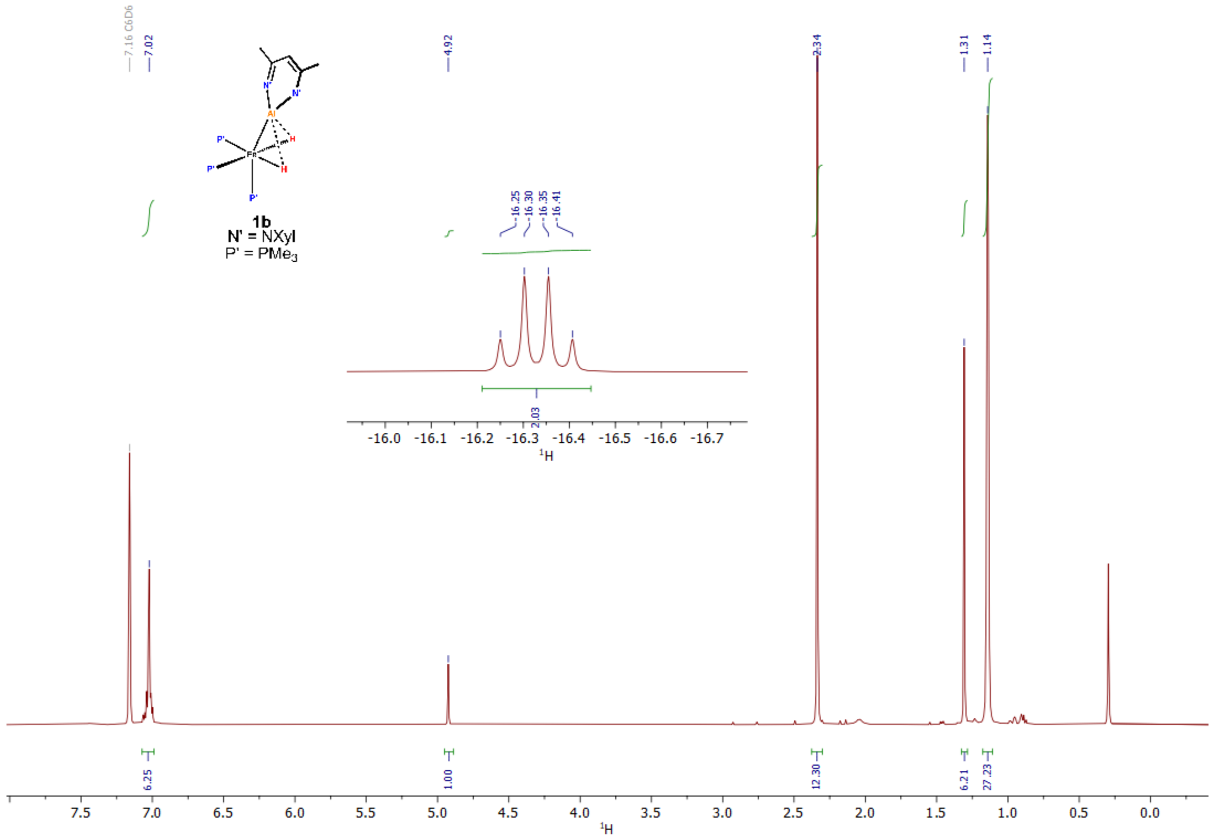

Figure S 8 ^{1}H NMR of **1b** (400 MHz, C_6D_6, 298 K)

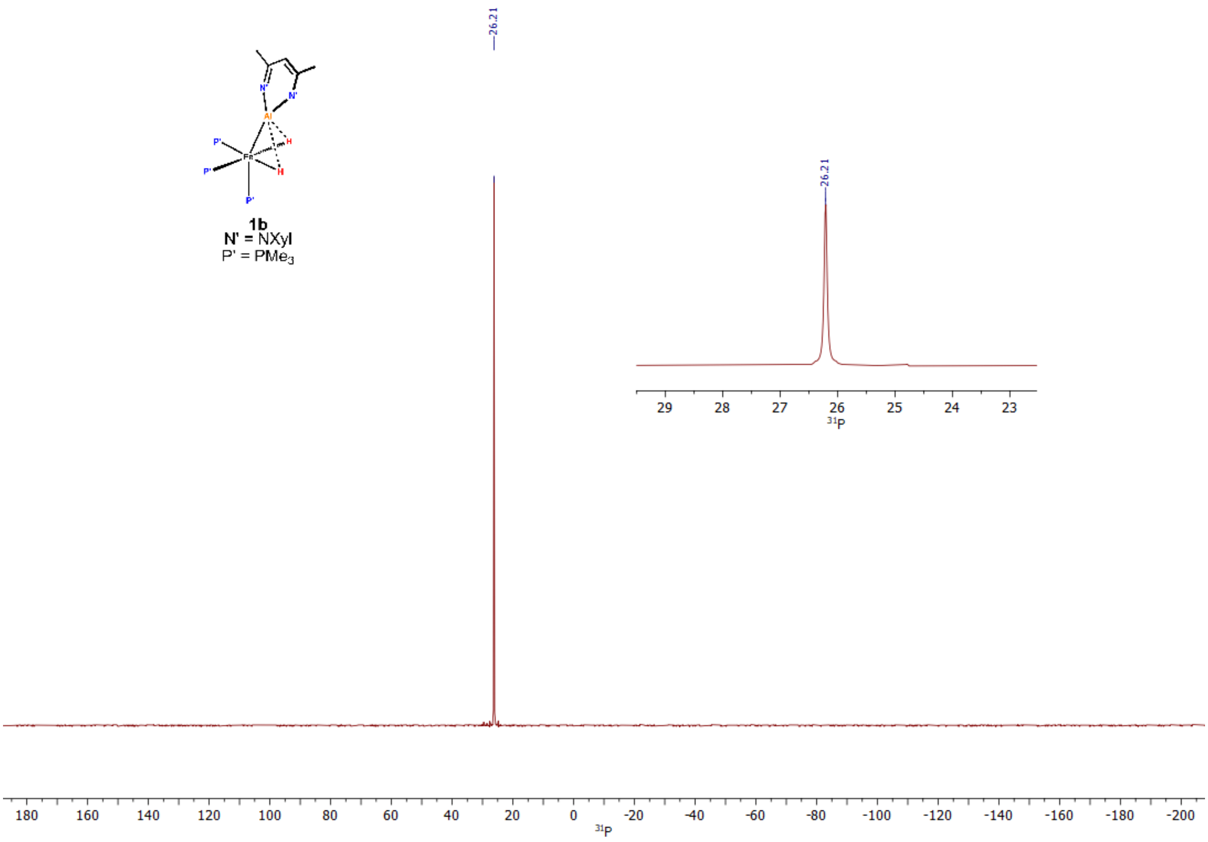

Figure S 9 ^{31}P{^{1}H} NMR of **1b** (162 MHz, C_6D_6, 298 K)

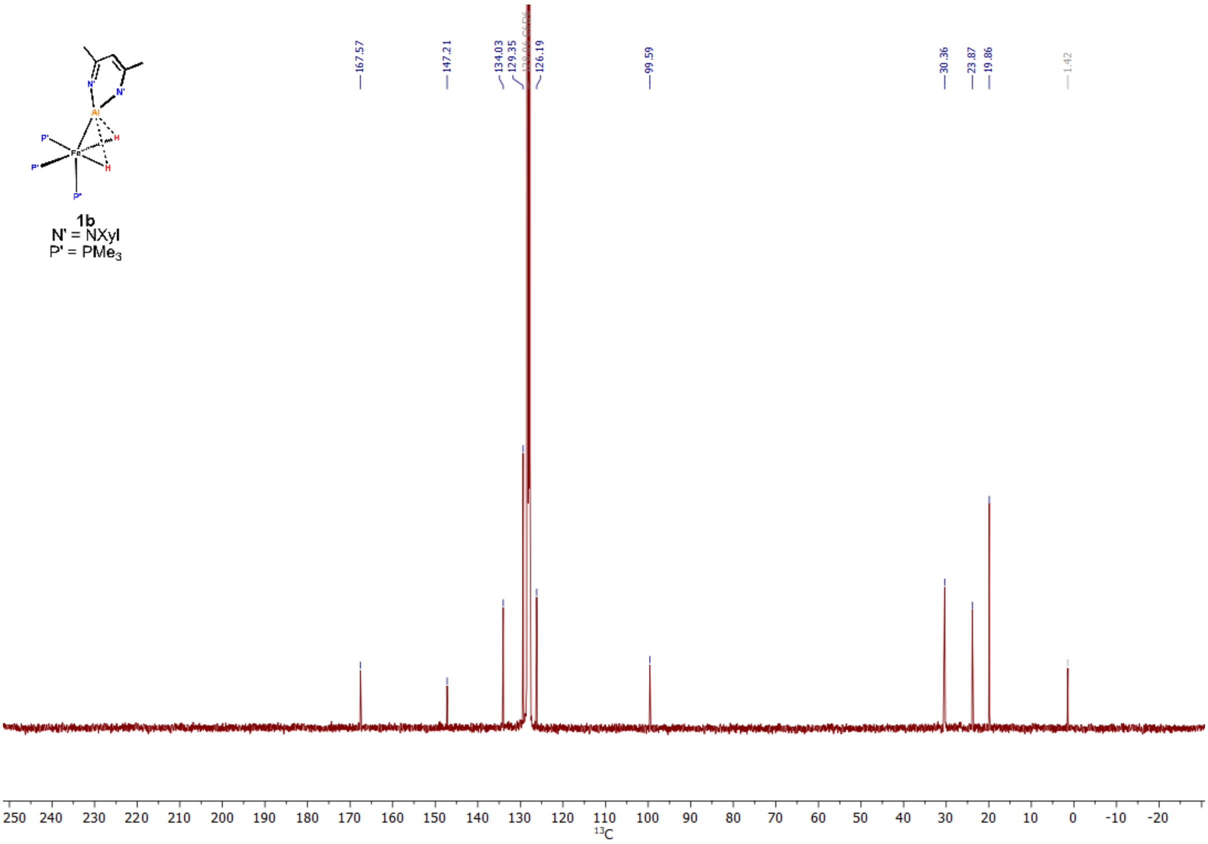

Figure S 10 ^{13}C{^{1}H} NMR of **1b** (101 MHz, C_6D_6, 298 K)

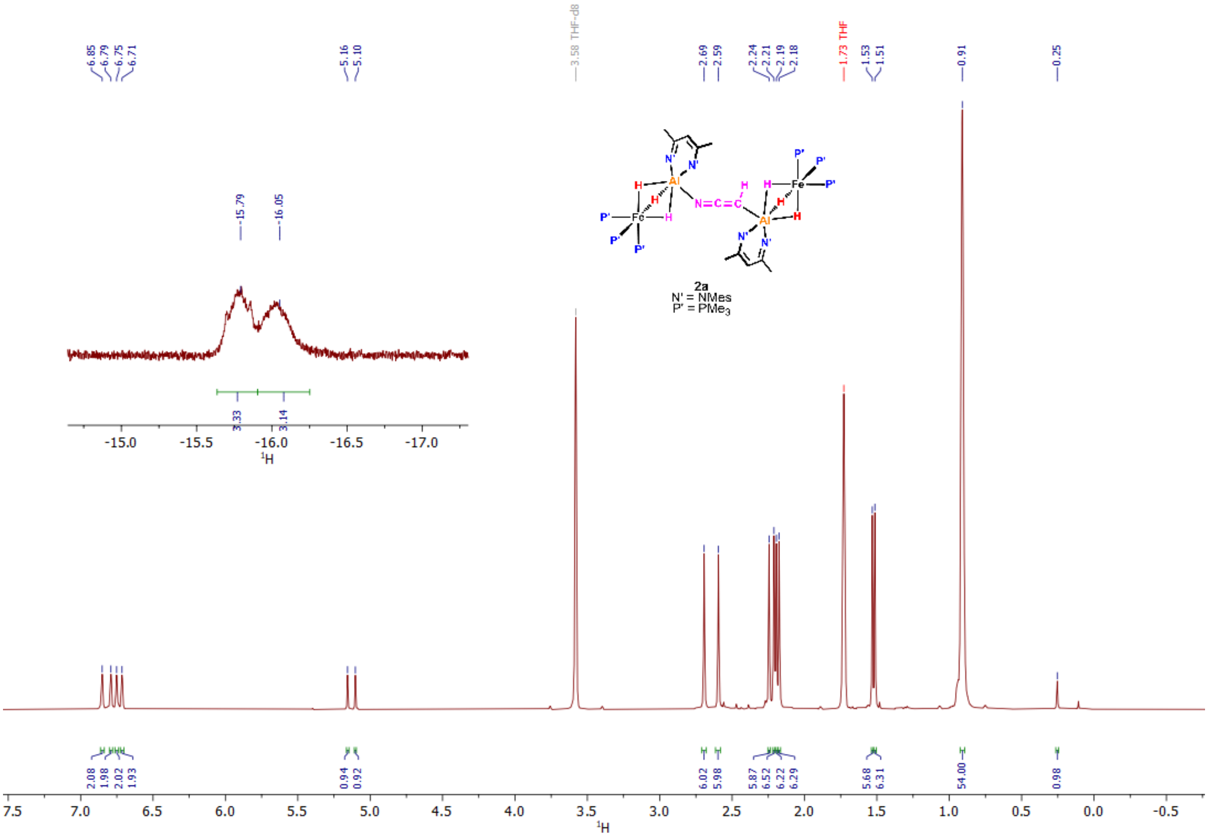

Figure S 11 ^{1}H NMR of **2a** (500 MHz, THF-[D_8], 298 K)

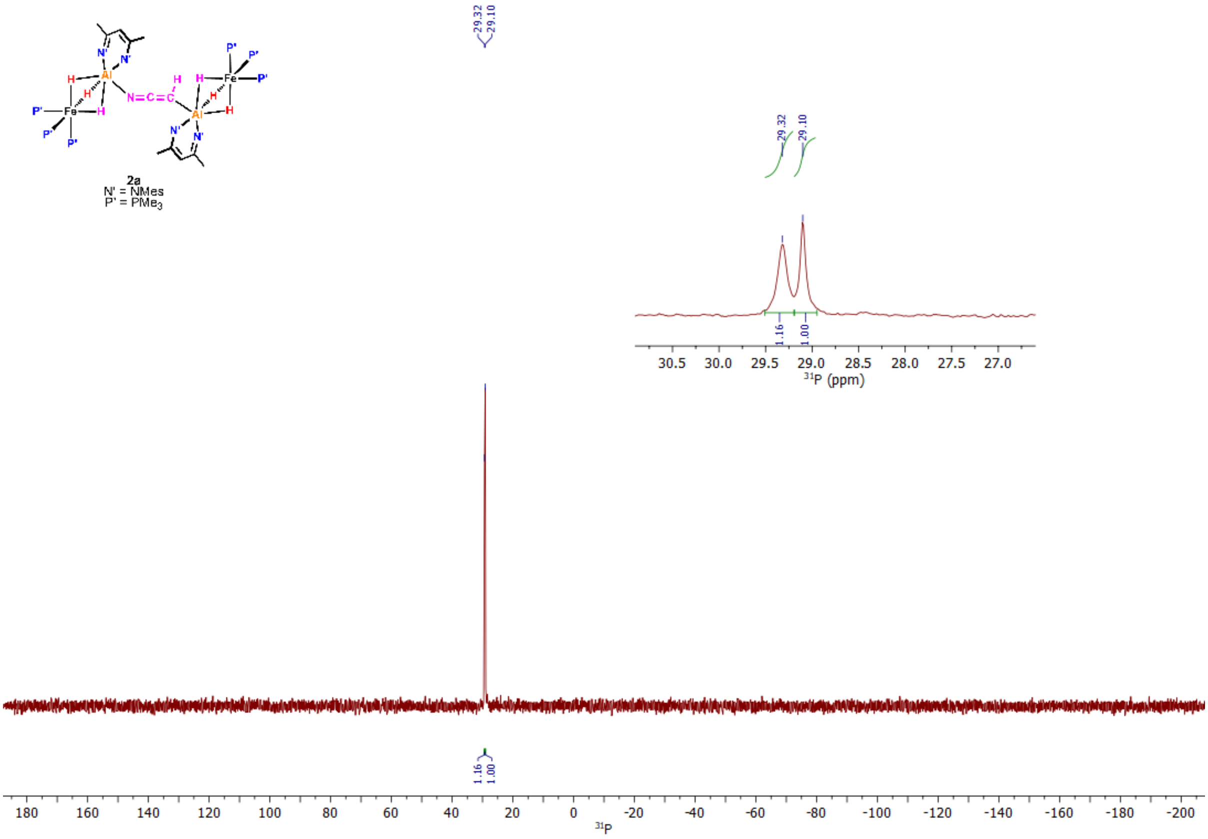

Figure S 12 ^{31}P{^{1}H} NMR of **2a** (202 MHz, THF-[D_8], 298 K)

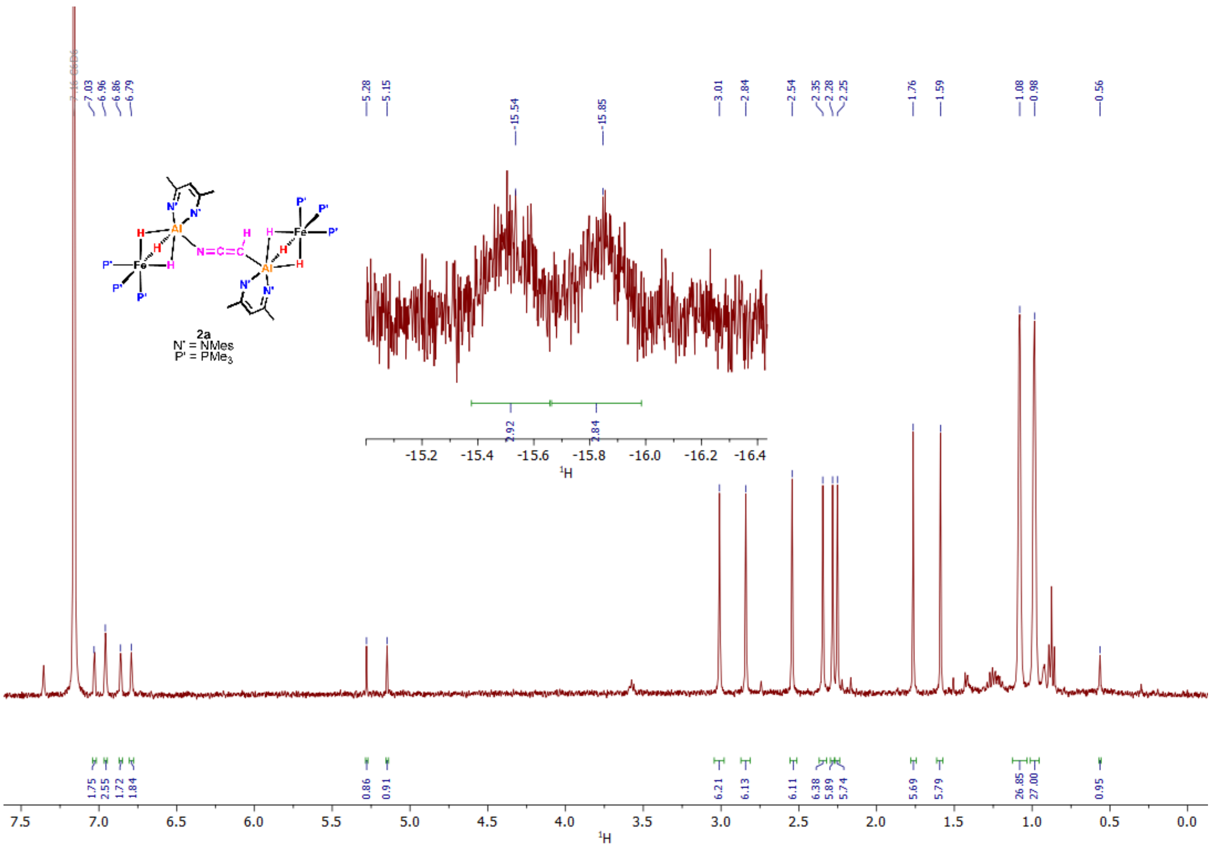

Figure S 13 ^{1}H NMR of **2a** (400 MHz, C_6D_6, 298 K)

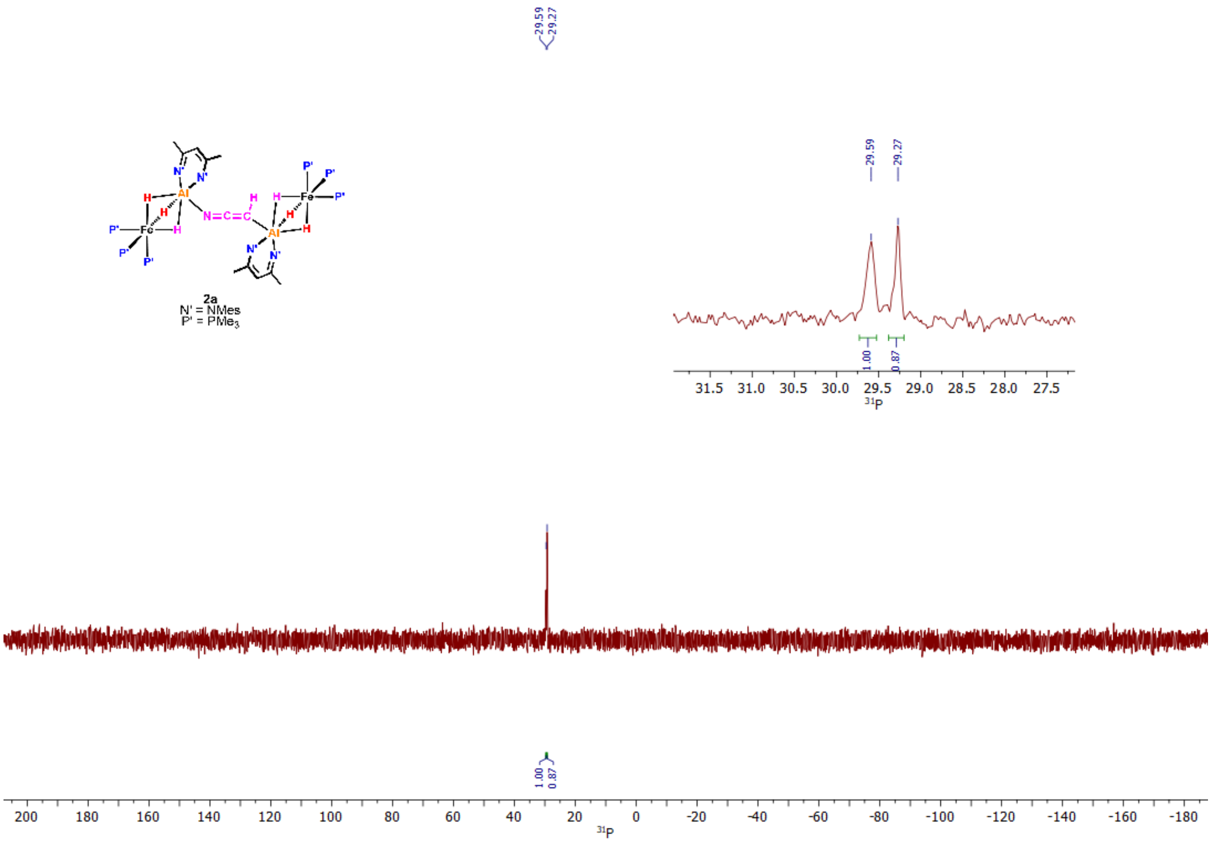

Figure S 14 $^{31}P\{^{1}H\}$ NMR of **2a** (162 MHz, C_6D_6, 298 K)

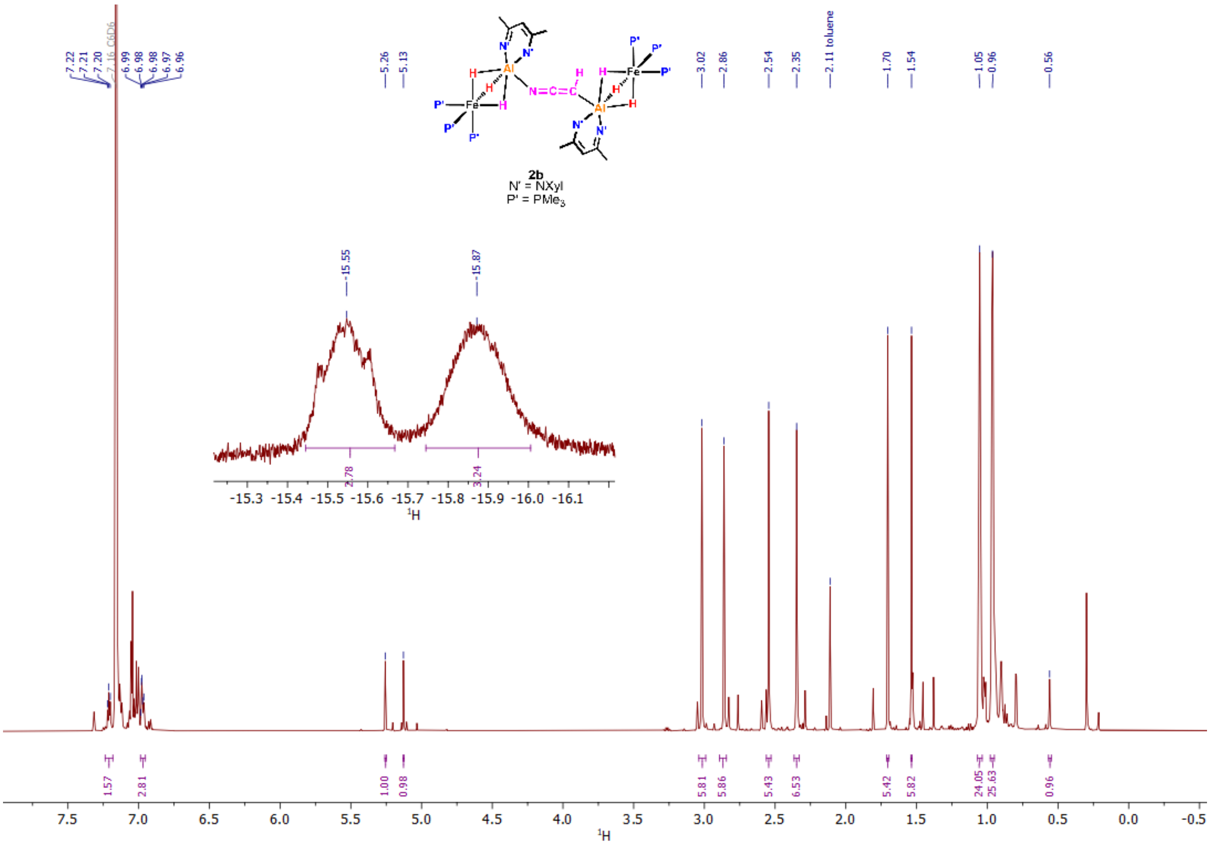

Figure S 15 ^{1}H NMR of **2b** (500 MHz, C_6D_6, 298 K)

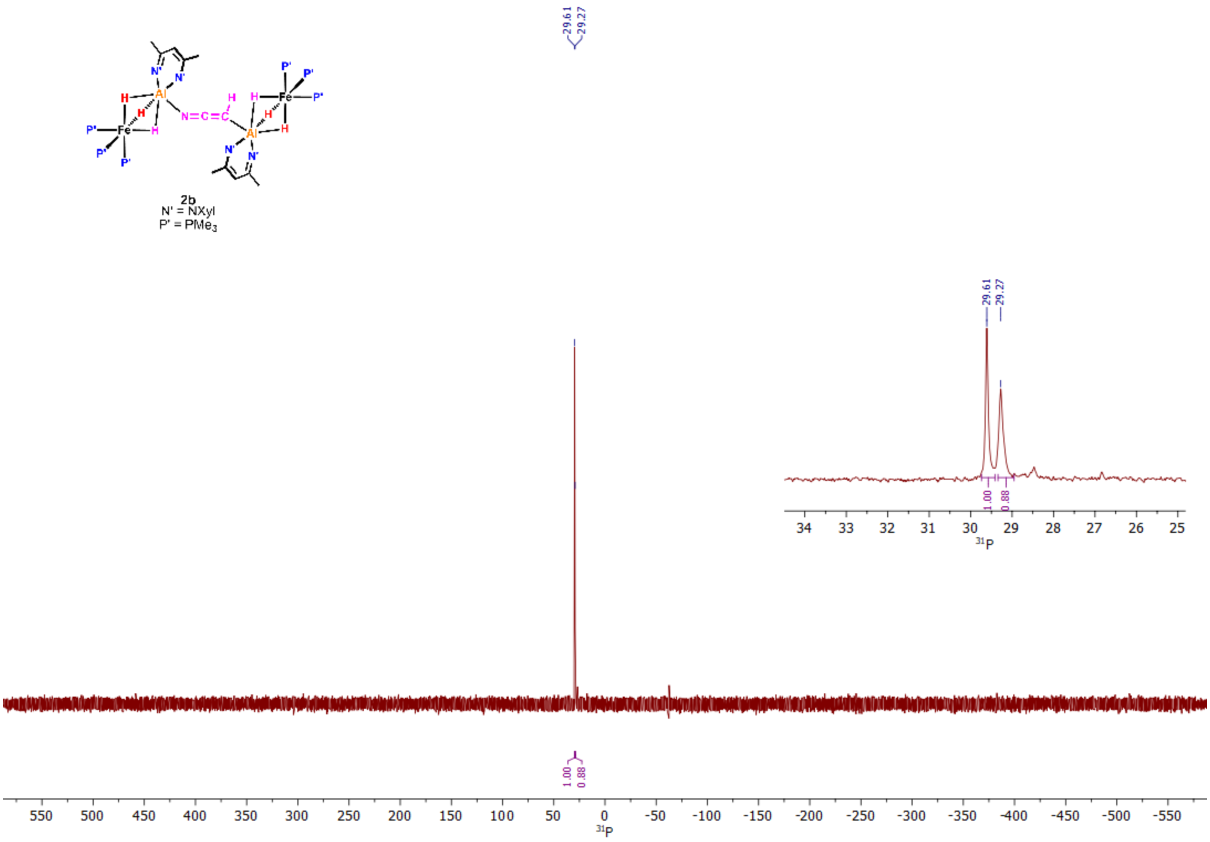

Figure S 16 $^{31}P\{^{1}H\}$ NMR of **2b** (202 MHz, C_6D_6, 298 K)

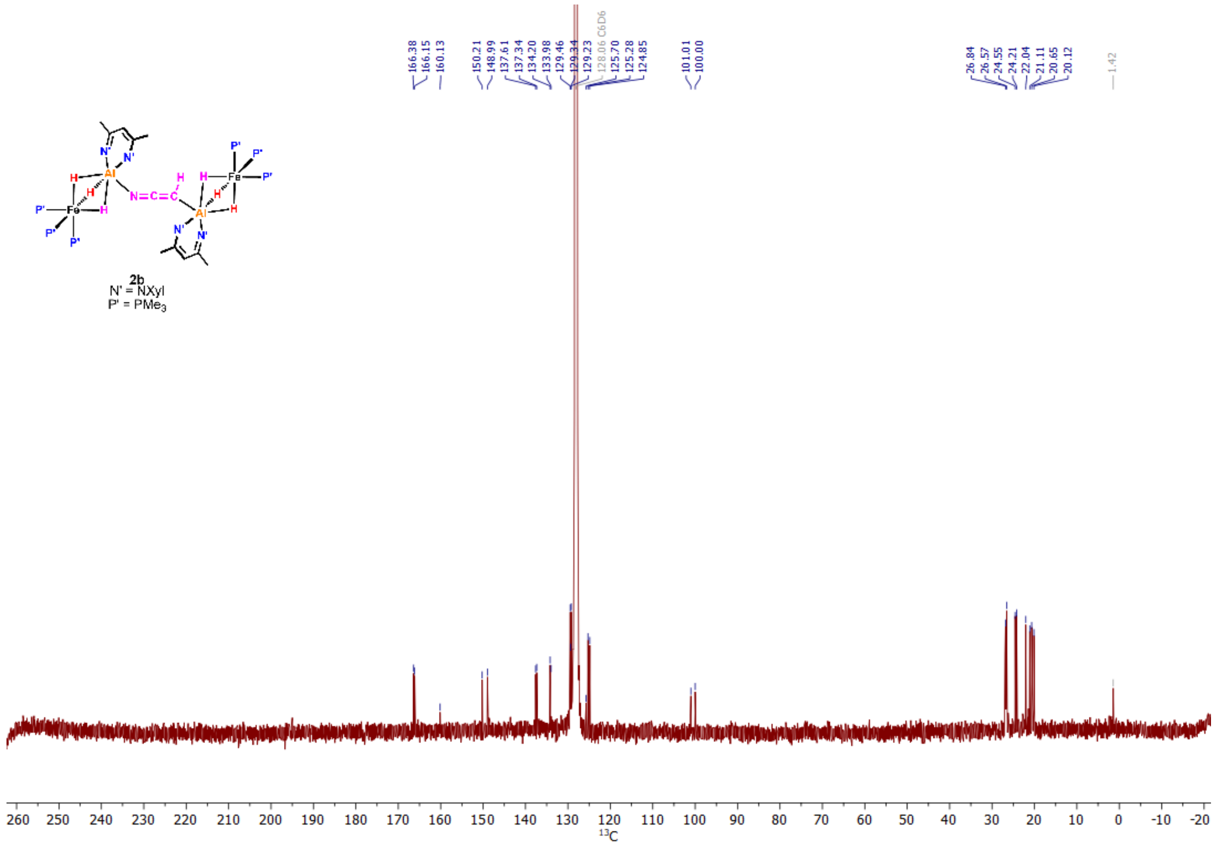

Figure S 17 $^{13}C\{^{1}H\}$ NMR of **2b** (125 MHz, C_6D_6, 298 K)

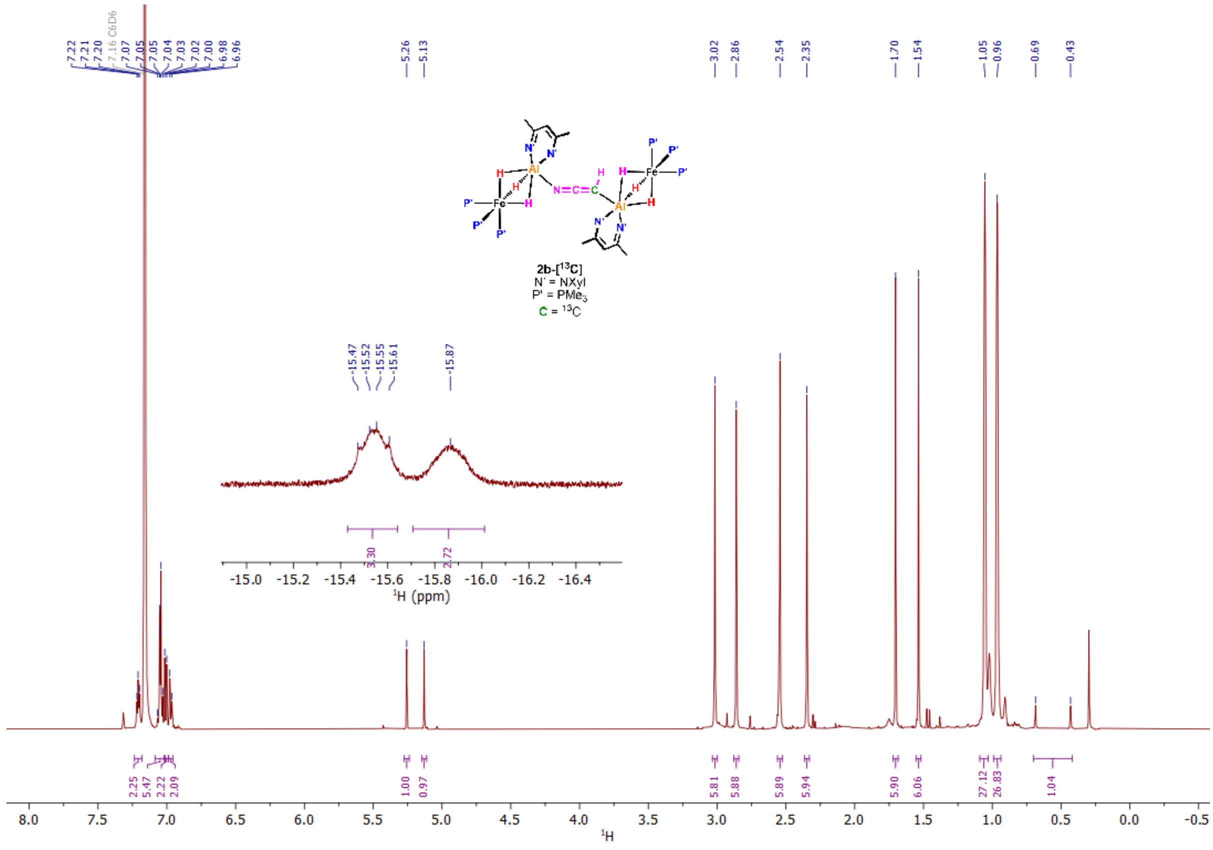

Figure S 18 ^{1}H NMR of **2b-[^{13}C]** (500 MHz, C_6D_6, 298 K)

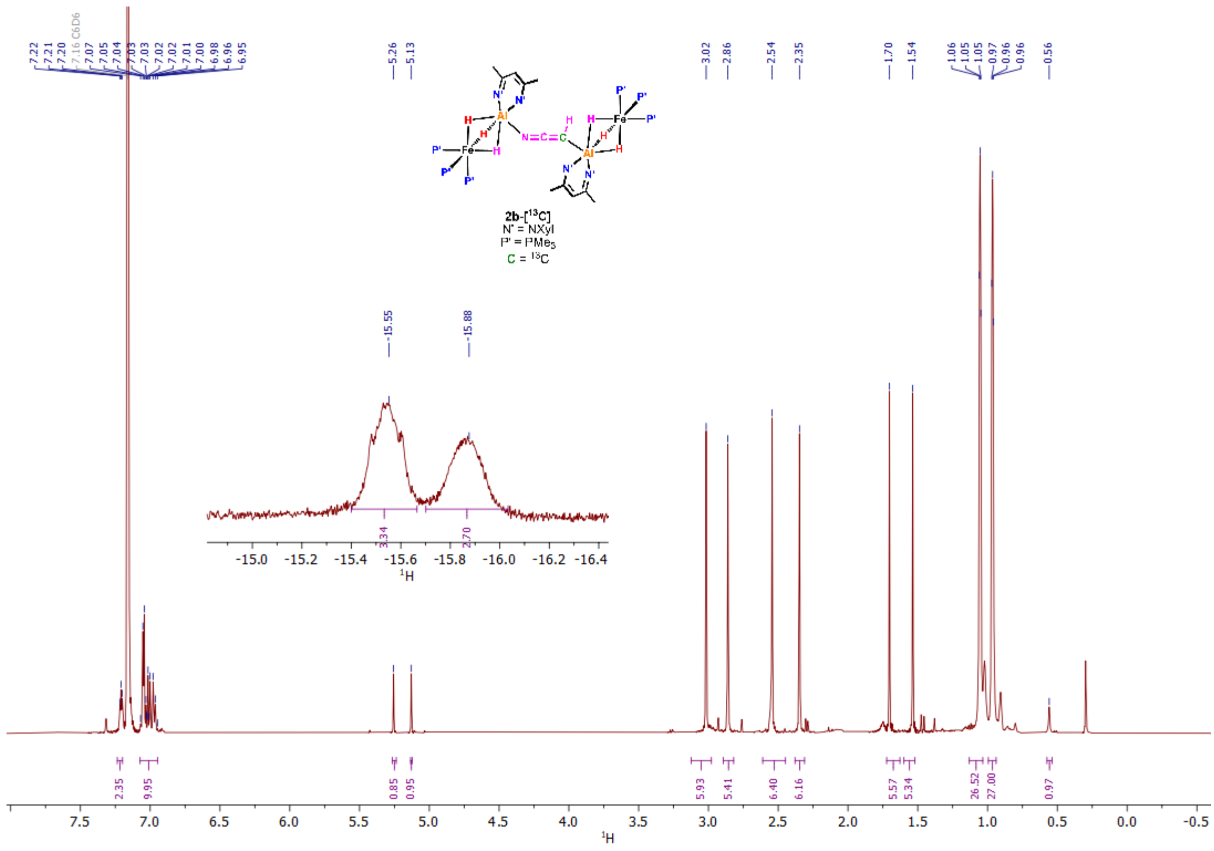

Figure S 19 $^{1}H\{^{13}C\}$ NMR of **2b-[^{13}C]** (500 MHz, C_6D_6, 298 K)

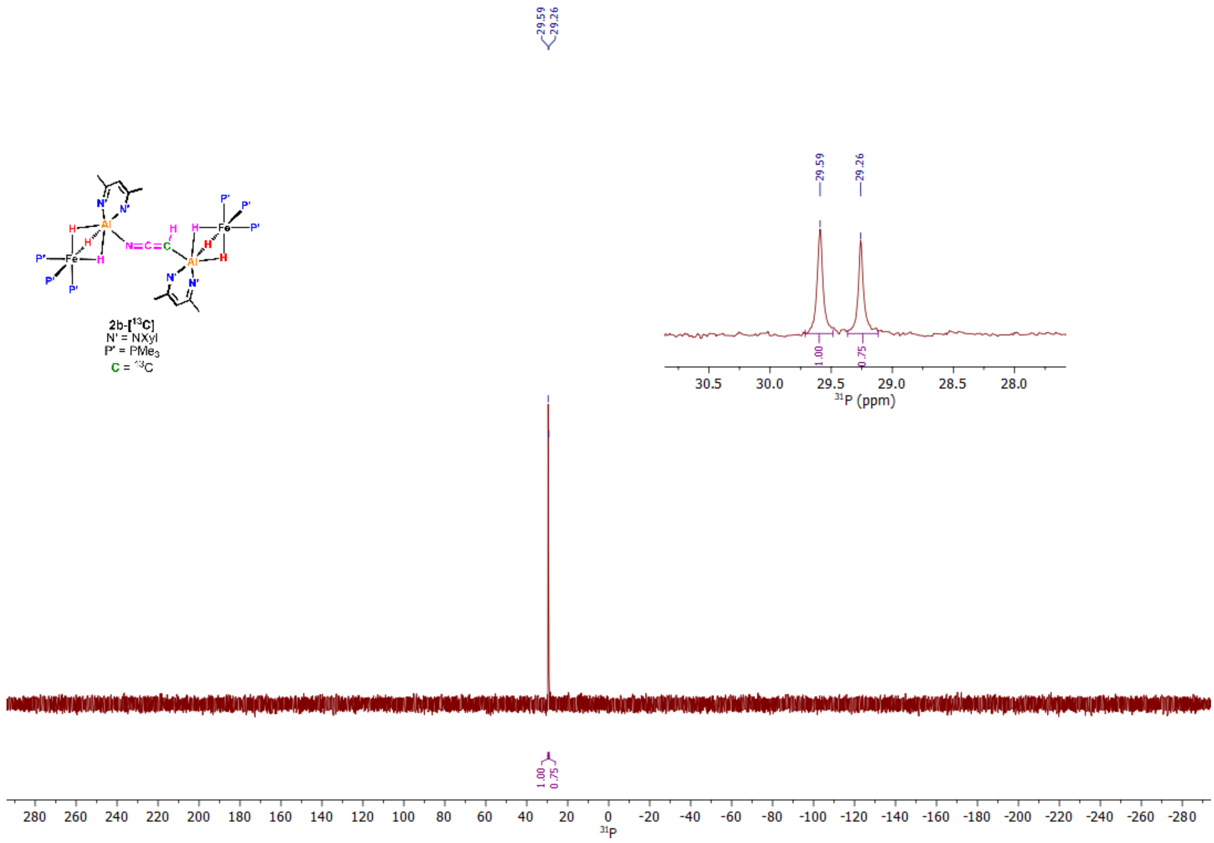

Figure S 20 $^{31}P\{^{1}H\}$ NMR of **2b-[^{13}C]** (202 MHz, C_6D_6, 298 K)

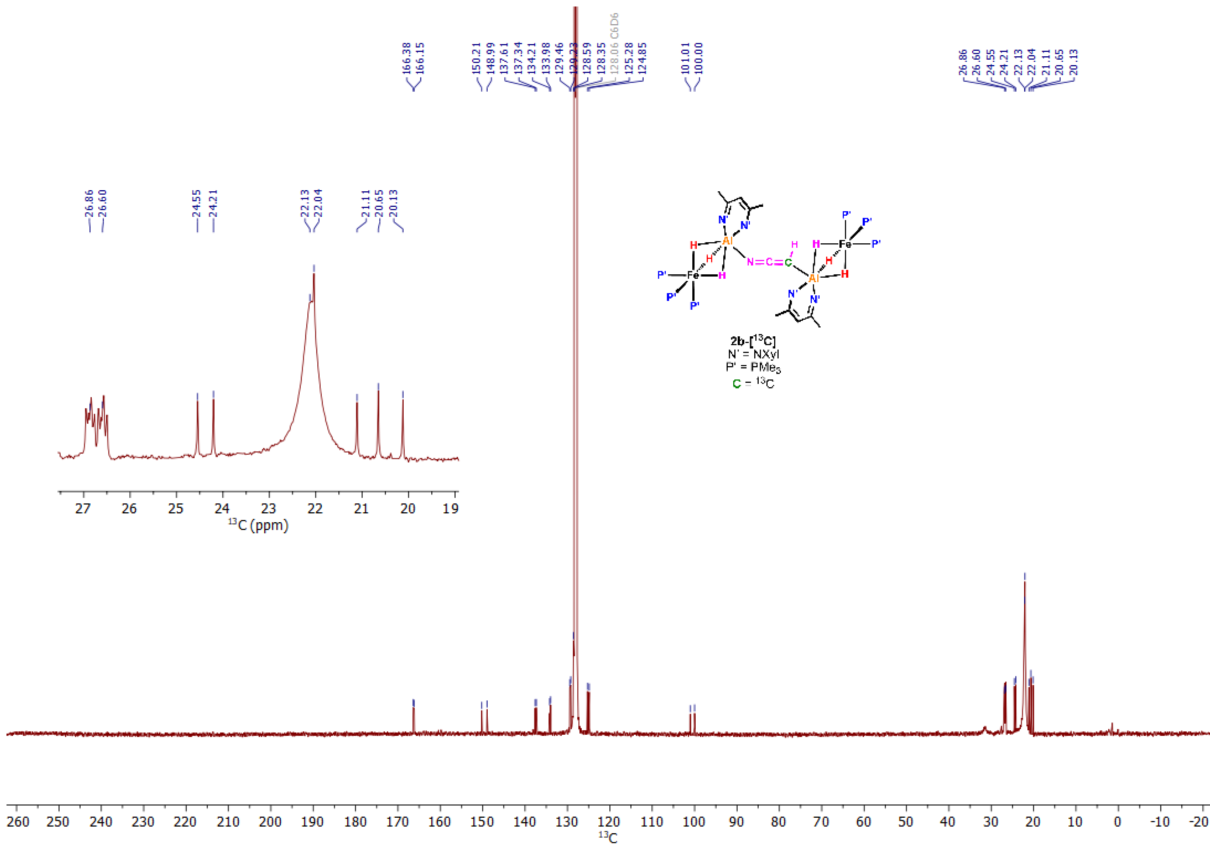

Figure S 21 $^{13}C\{^{1}H\}$ NMR of **2b-[^{13}C]** (125 MHz, C_6D_6, 298 K)

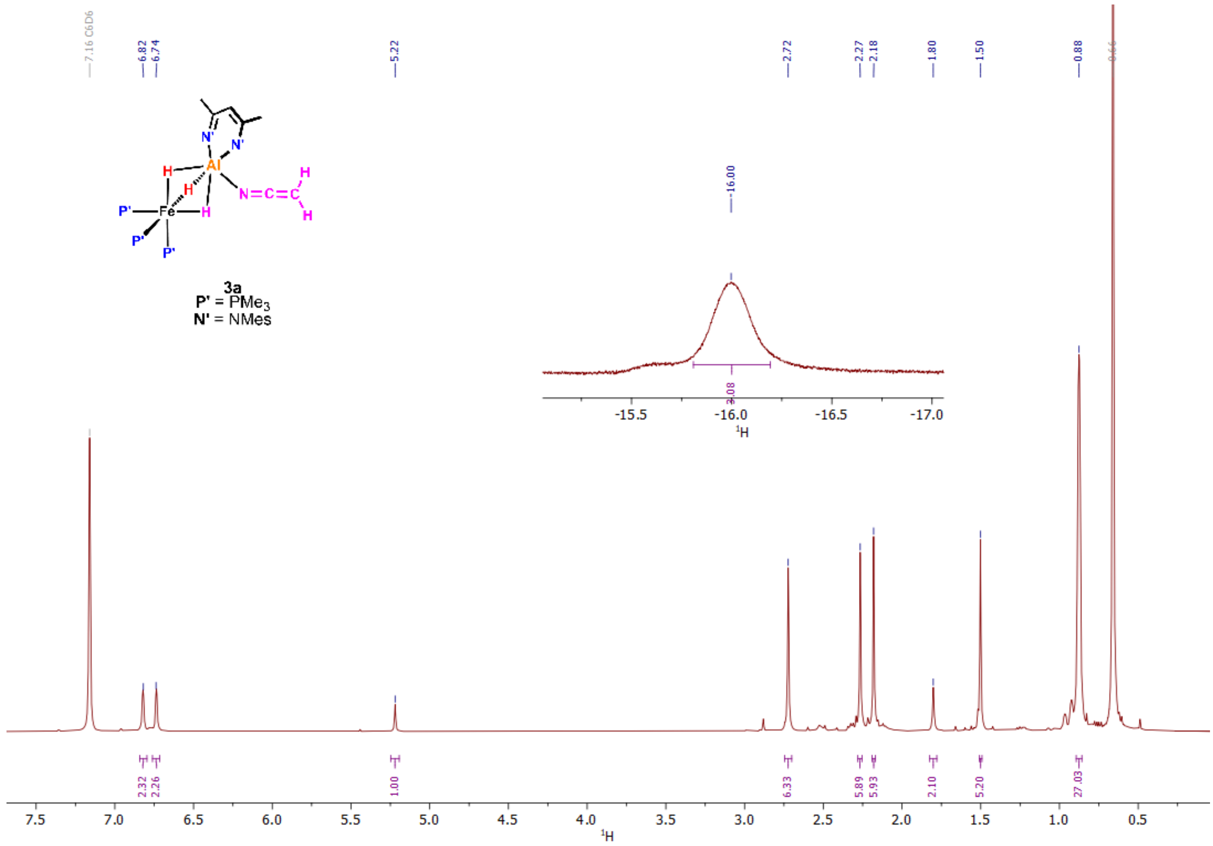

Figure S 22 ^{1}H NMR of **3a** (400 MHz, C_6D_6, 298 K)

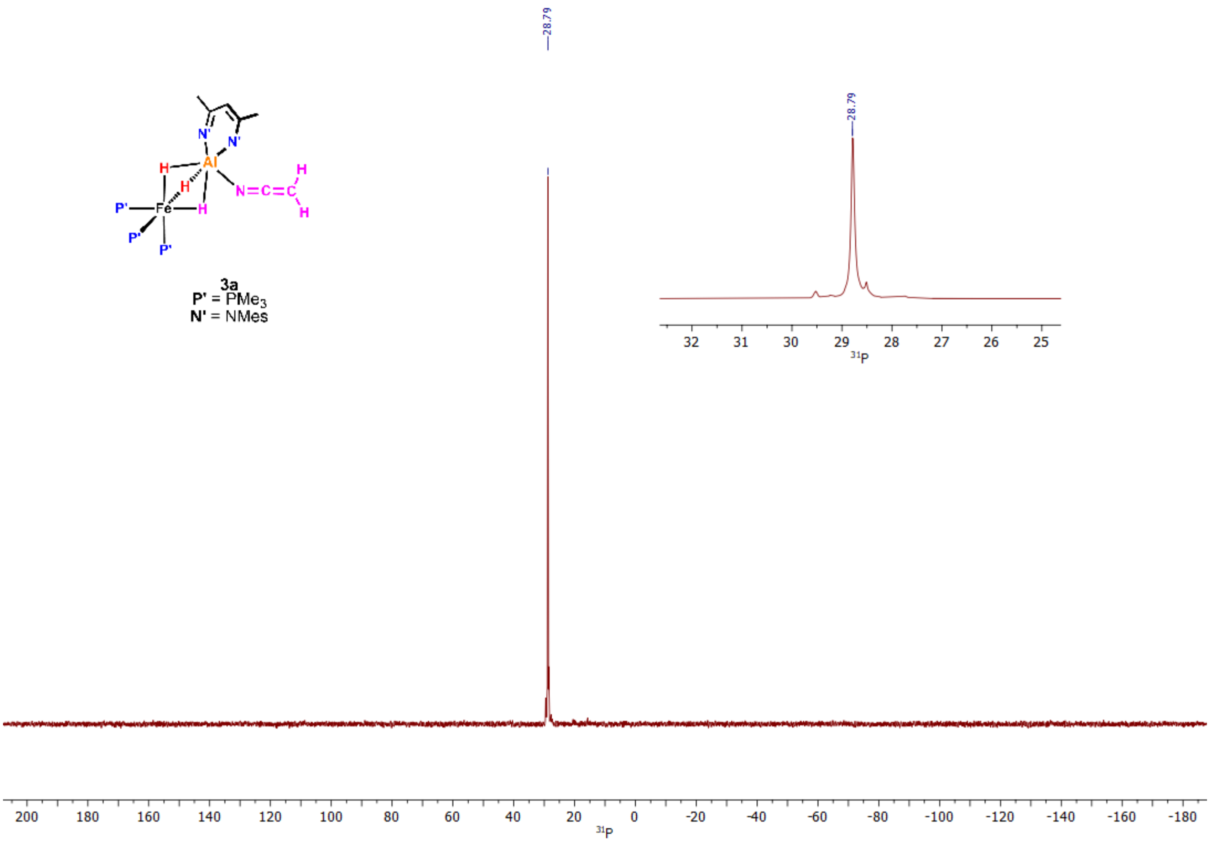

Figure S 23 $^{31}P\{^{1}H\}$ NMR of **3a** (162 MHz, C_6D_6, 298 K)

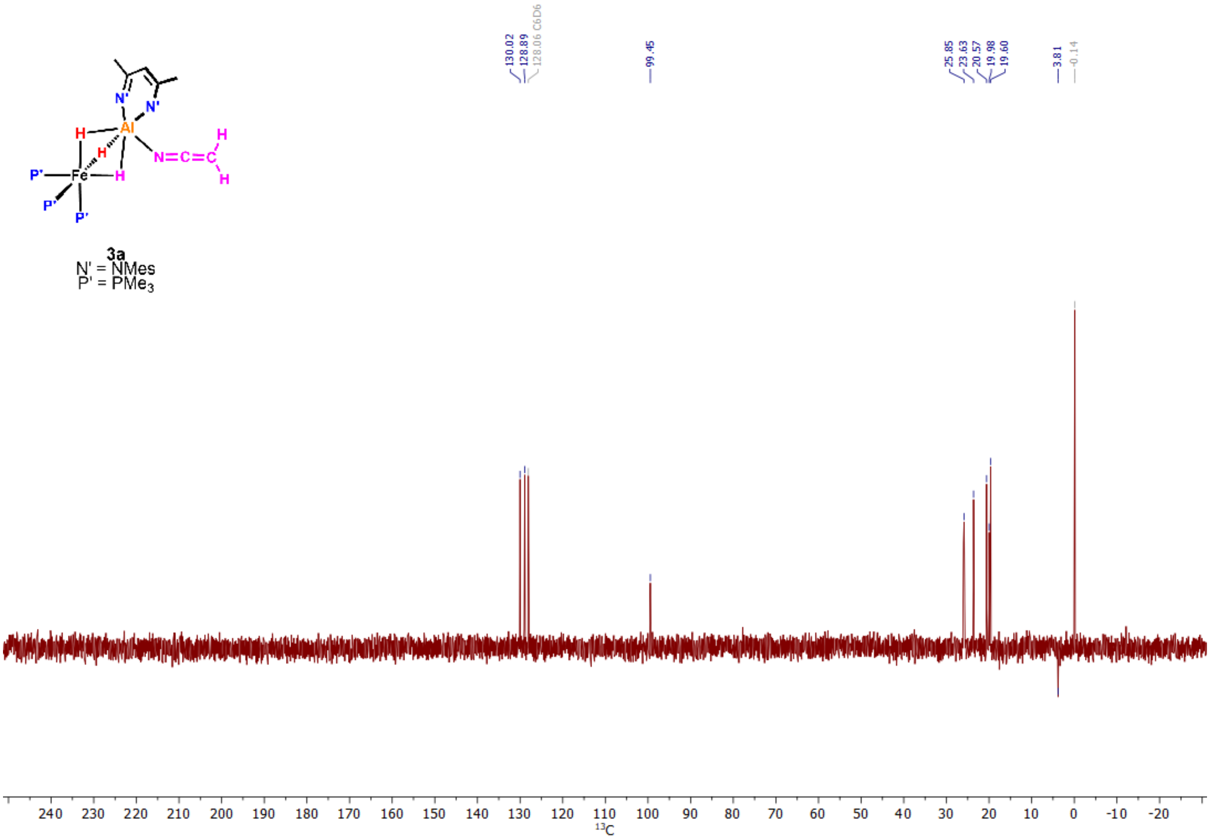

Figure S 24 DEPT-135 NMR of **3a** (101 MHz, C_6D_6, 298 K)

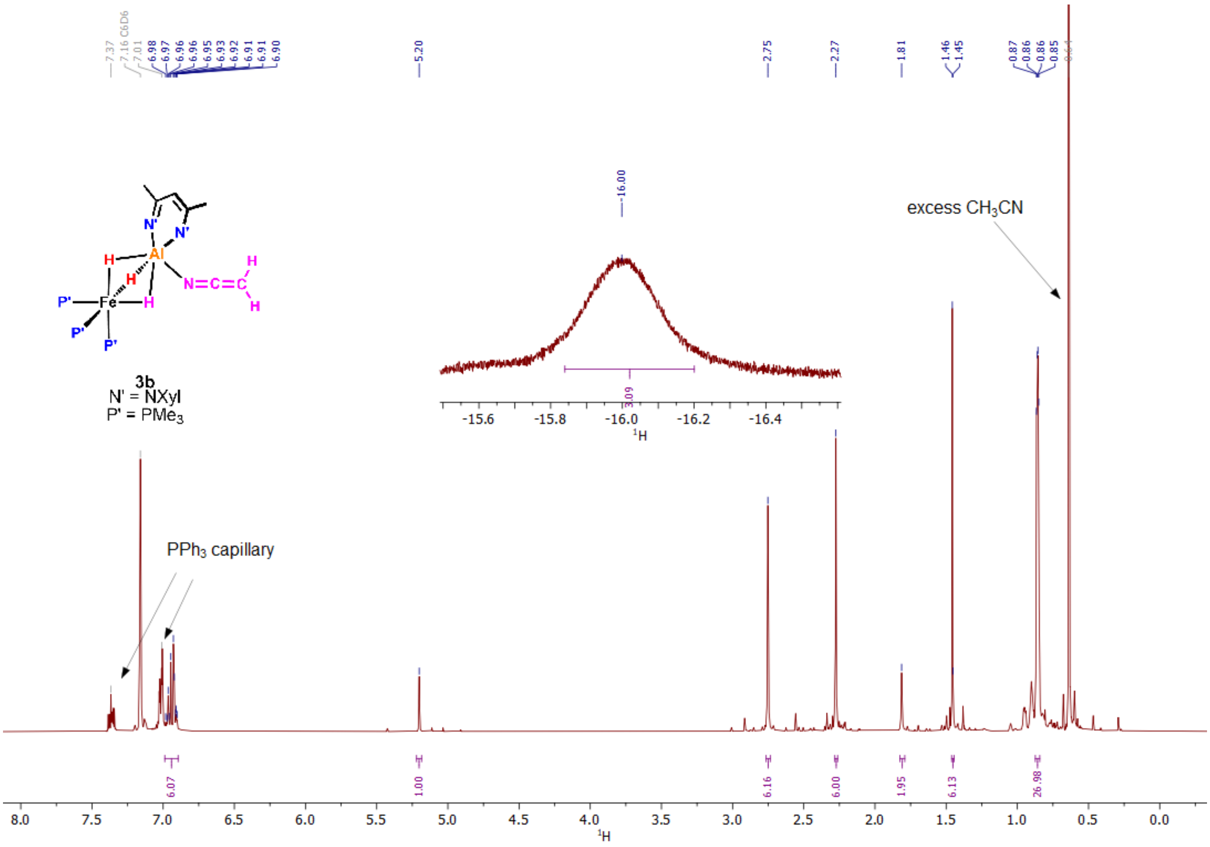

Figure S 25 ^{1}H NMR of **3b** (400 MHz, C_6D_6, 298 K)

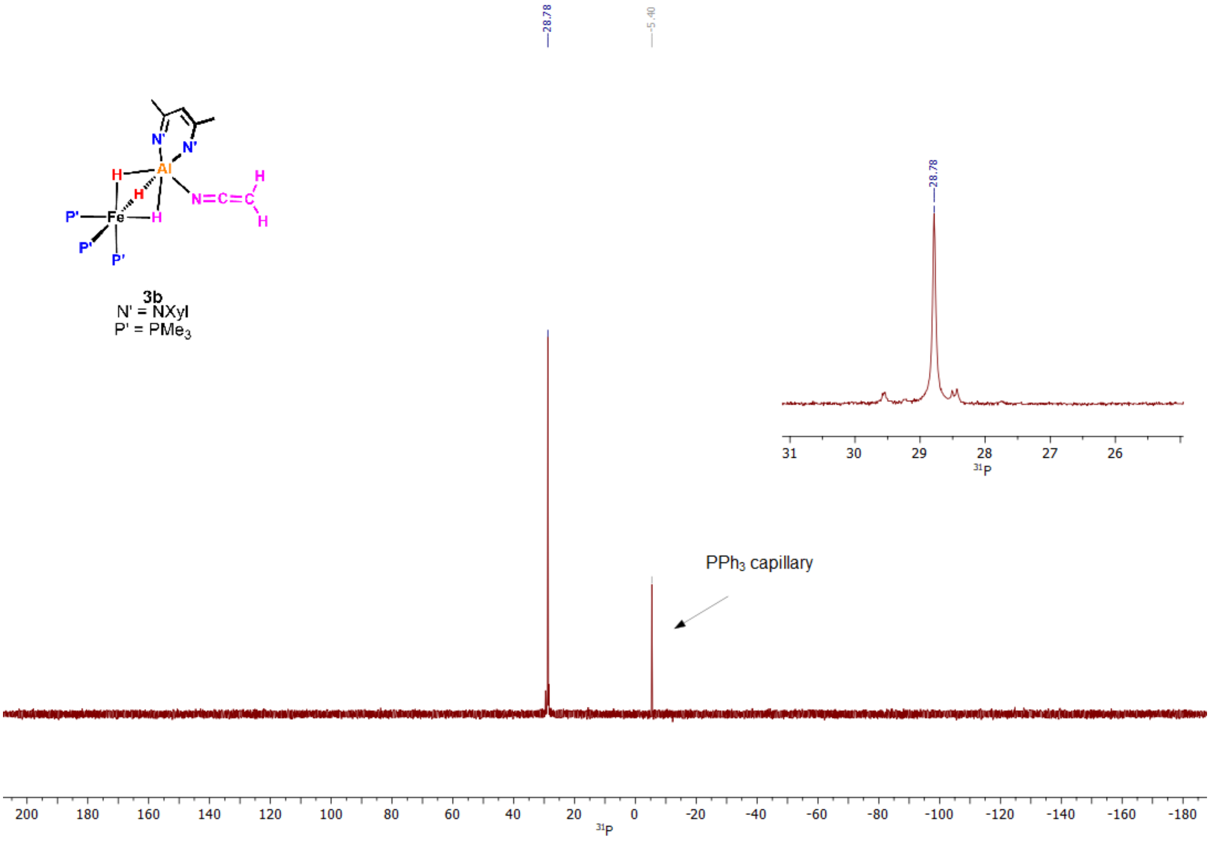

Figure S 26 $^{31}P\{^{1}H\}$ NMR of **3b** (400 MHz, C_6D_6, 298 K)

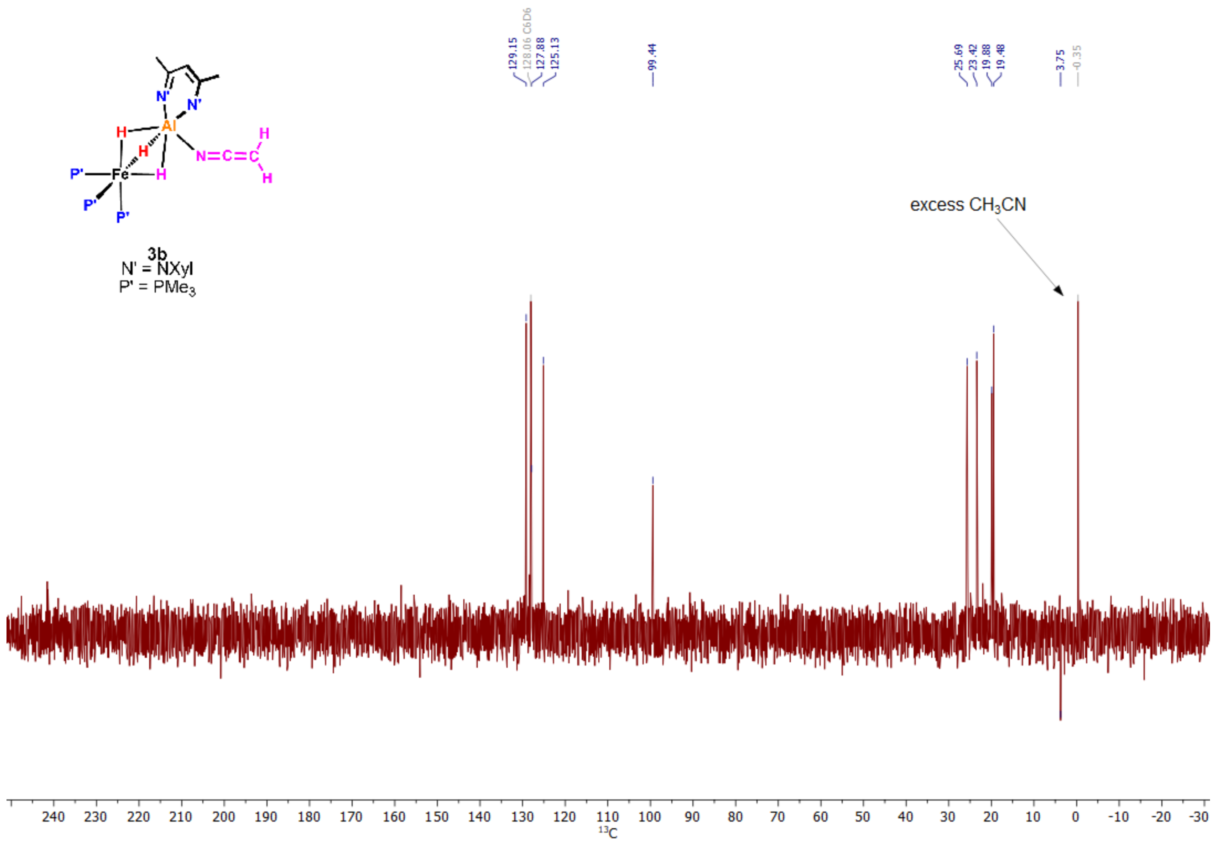

Figure S 27 DEPT-135 NMR of **3b** (400 MHz, C_6D_6, 298 K)

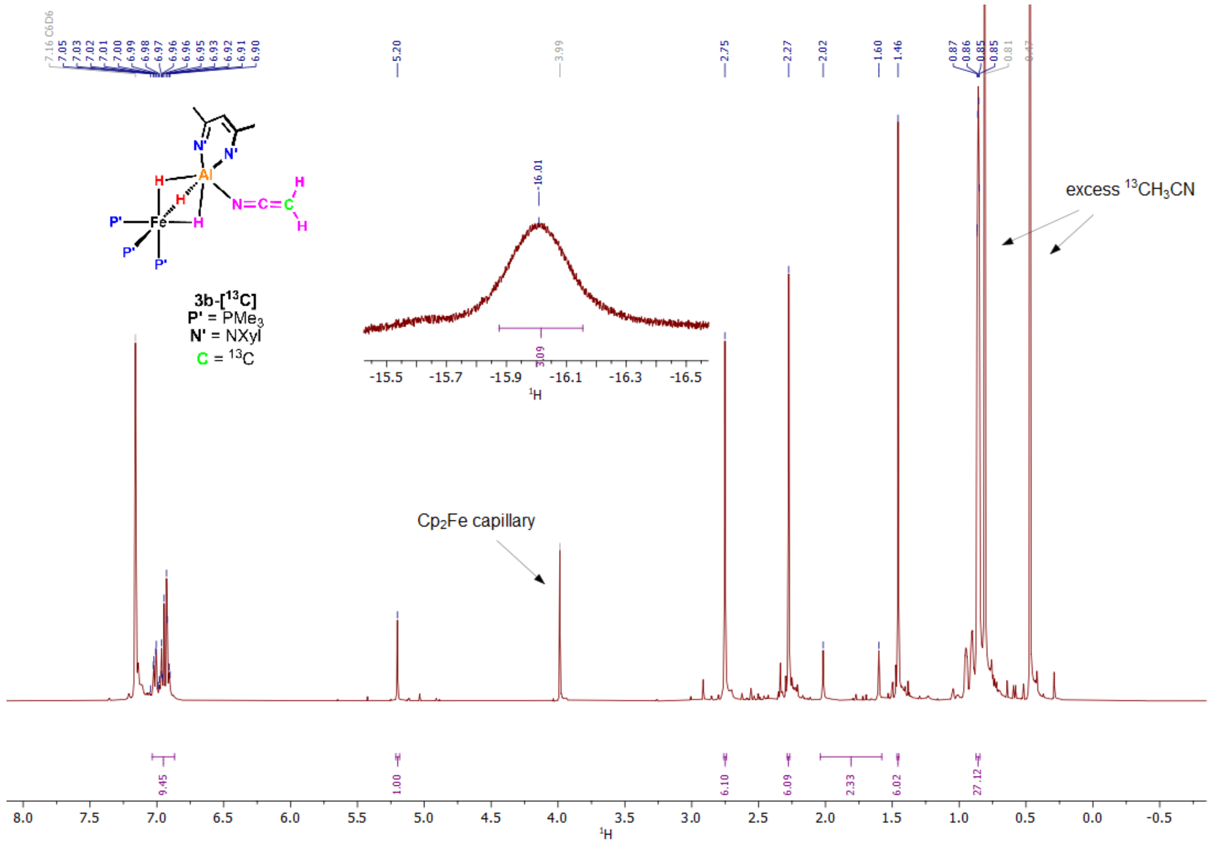

Figure S 28 ^{1}H NMR of **3b-[^{13}C]** (400 MHz, C_6D_6, 298 K)

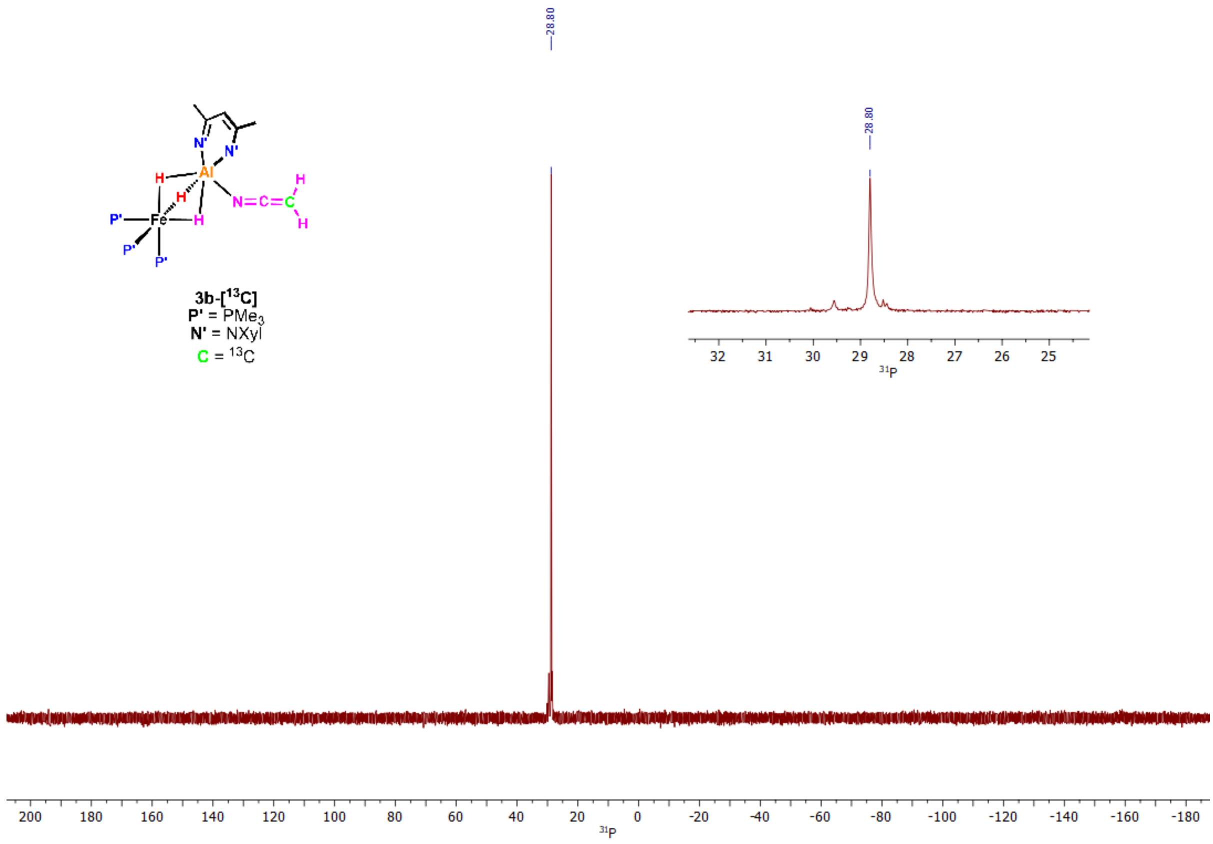

Figure S 29 $^{31}P\{^{1}H\}$ NMR of **3b-[^{13}C]** (162 MHz, C_6D_6, 298 K)

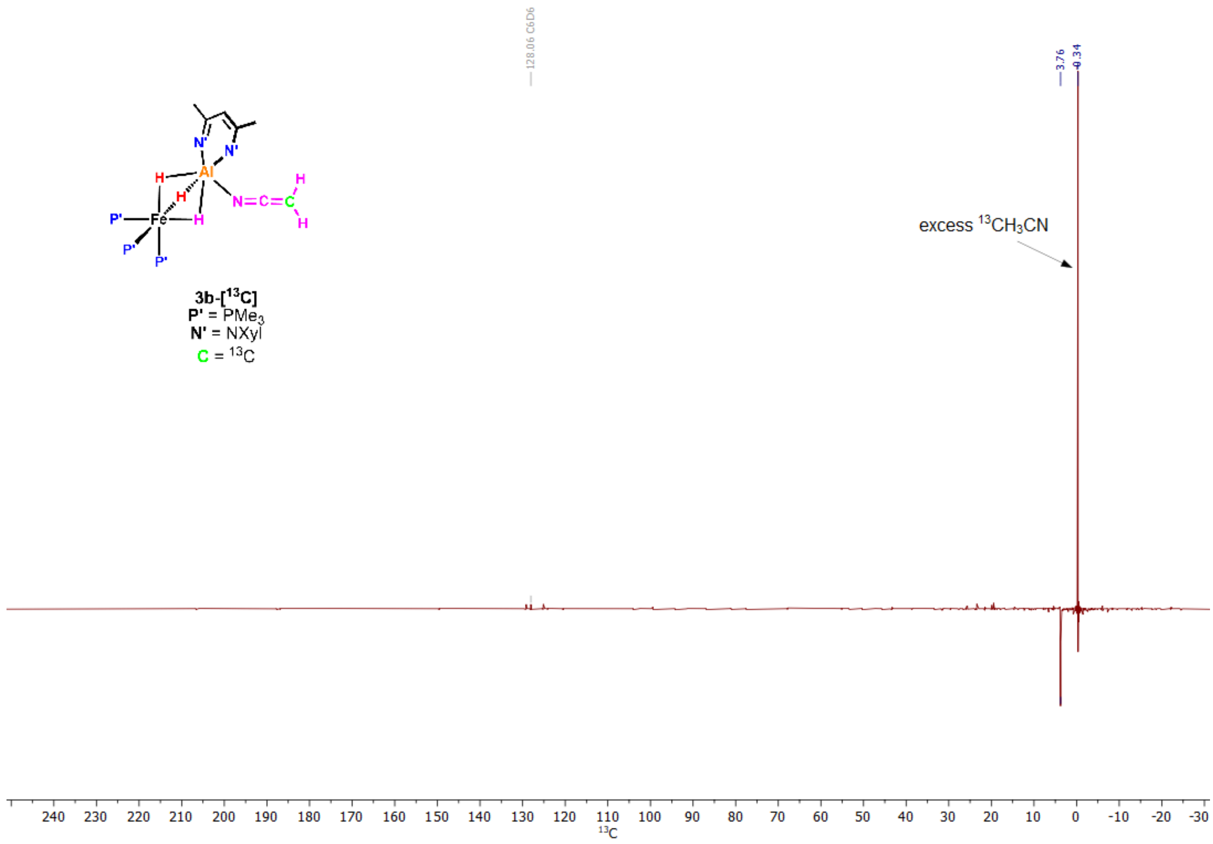

Figure S 30 DEPT-135 NMR of **3b-[^{13}C]** (101 MHz, C_6D_6, 298 K)

7 Selected IR spectra

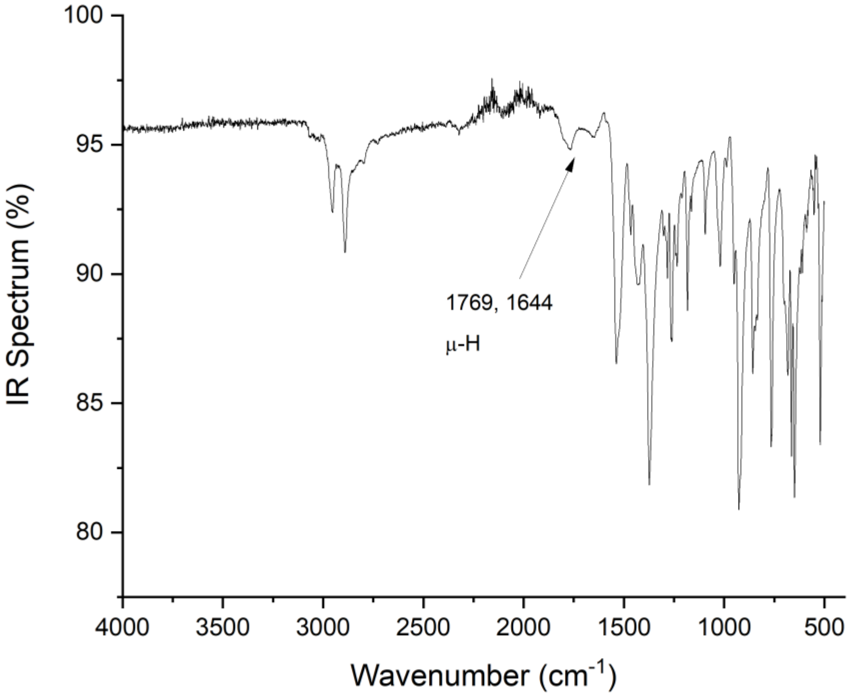

Figure S 31 ATR IR spectra of **1b** (298 K).

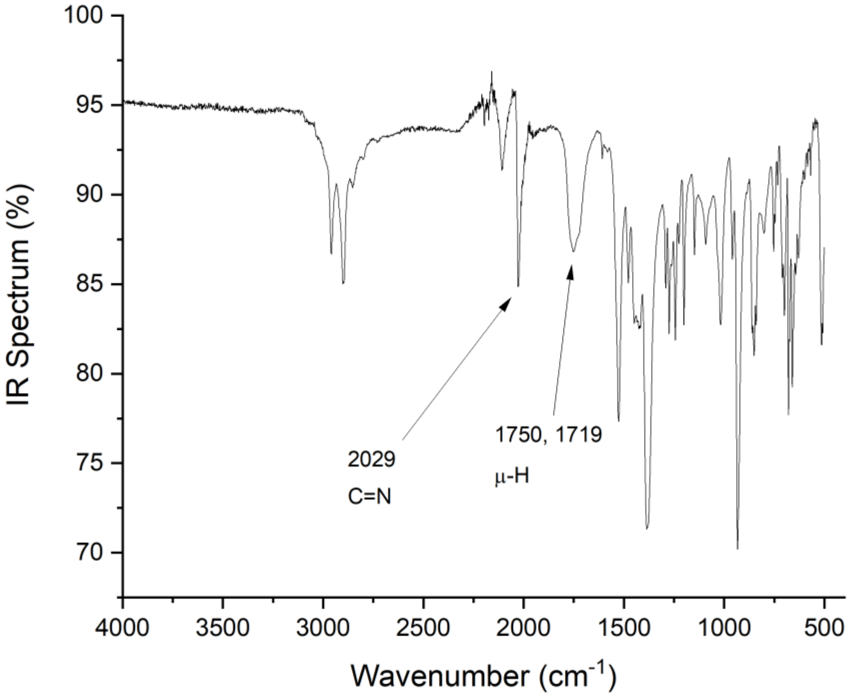

Figure S 32 ATR IR spectra of **2a** (298 K).

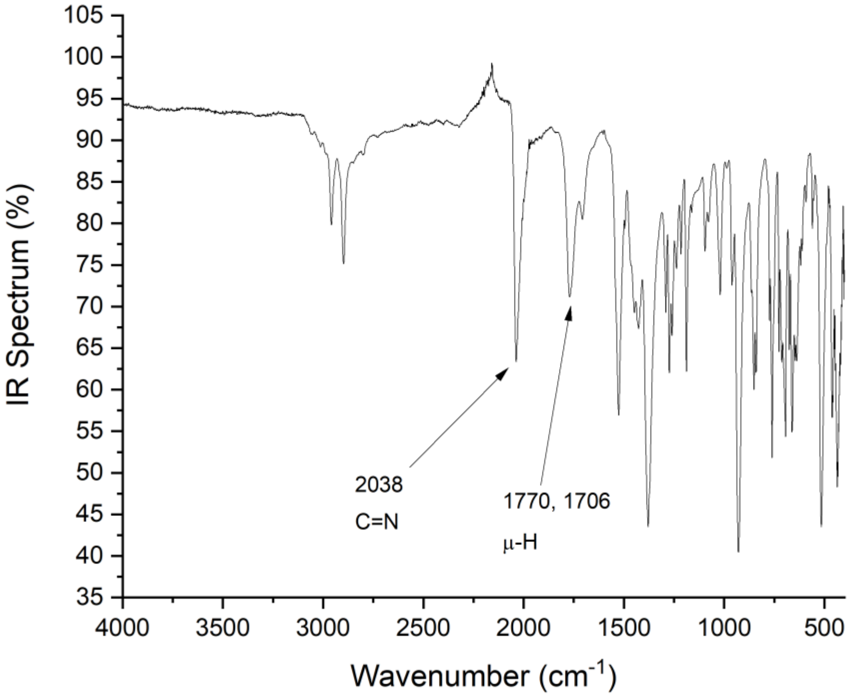

Figure S 33 ATR IR spectra of **2b** (298 K).

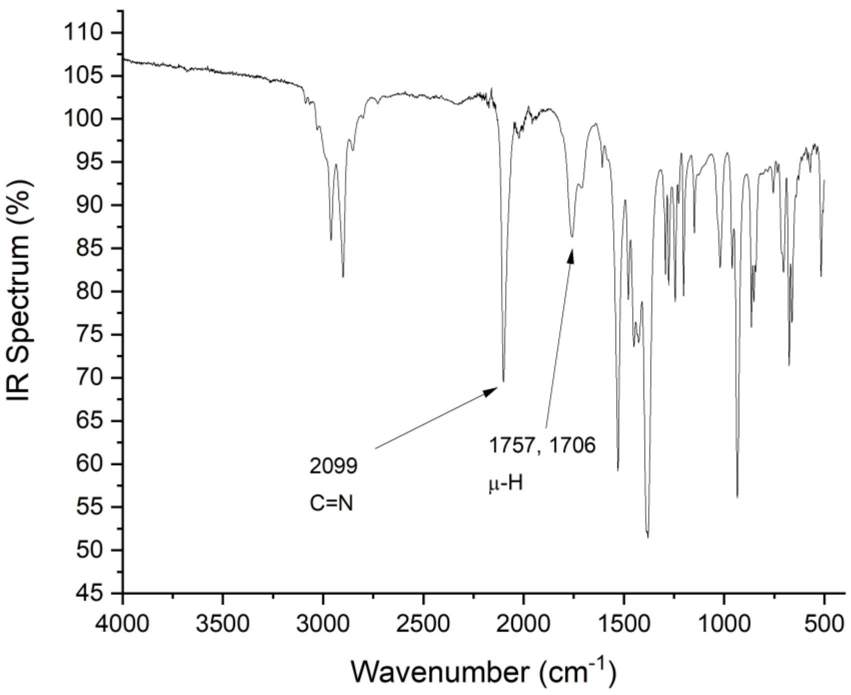

Figure S 34 ATR IR spectra of **3a** (298 K).

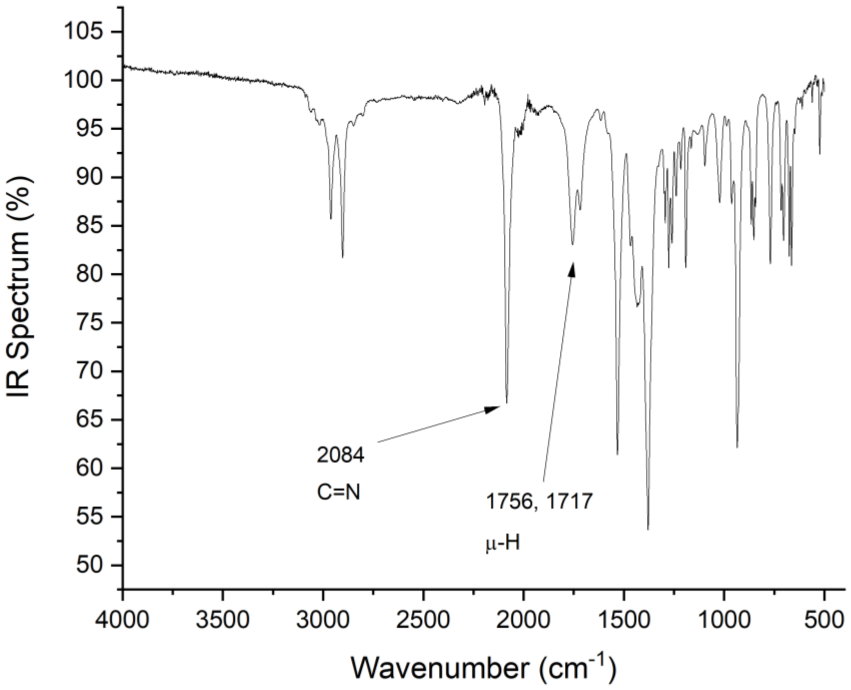

Figure S 35 ATR IR spectra of **3b** (298 K).

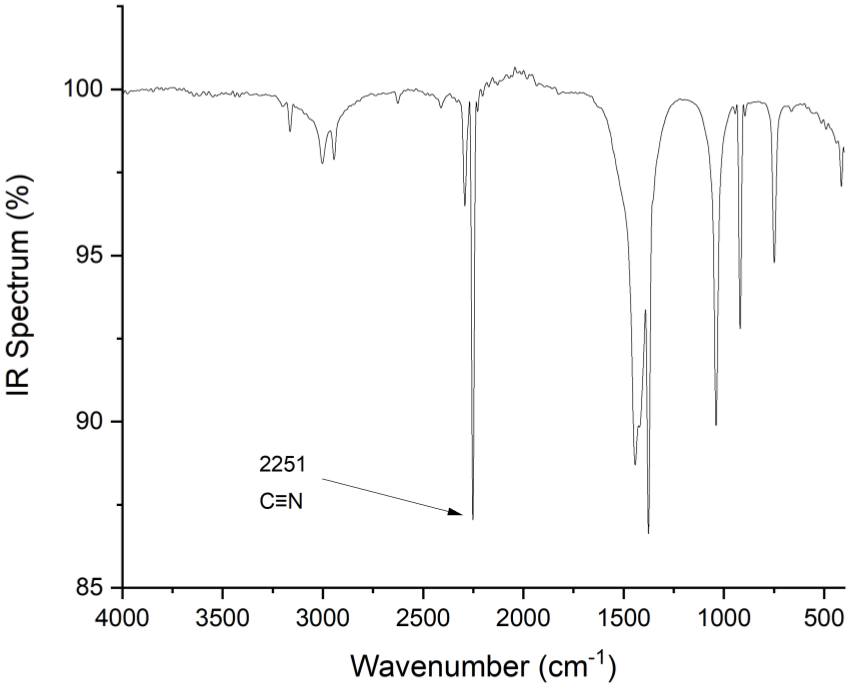

Figure S 36 ATR IR spectra of CH_3CN (298 K).

8 References

[1] S. Yow, S. J. Gates, A. J. P. White, M. R. Crimmin, *Angew. Chemie Int. Ed.* **2012**, *51*, 12559–12563.

[2] N. Gorgas, A. J. P. White, M. R. Crimmin, *J. Am. Chem. Soc.* **2022**, *144*, 8770–8777.

[3] O. V. Dolomanov, L. J. Bourhis, R. J. Gildea, J. A. K. Howard, H. Puschmann, *J. Appl. Crystallogr.* **2009**, *42*, 339–341.

[4] Bruker AXS, **1998**.

[5] G. M. Sheldrick, *Acta Crystallogr. Sect. A Found. Adv.* **2015**, *71*, 3–8.

[6] G. M. Sheldrick, *Acta Crystallogr. Sect. C Struct. Chem.* **2015**, *71*, 3–8.

[7] D. J. Frisch, M. J.; Trucks, G. W.; Schlegel, H. B.; Scuseria, G. E.; Robb, M. A.; Cheeseman, J. R.; Scalmani, G.; Barone, V.; Mennucci, B.; Petersson, G. A.; Nakatsuji, H.; Caricato, M.; Li, X.; Hratchian, H. P.; Izmaylov, A. F.; Bloino, J.; Zheng, G.; Sonnenb, **2009**.

[8] E. D. Glendening, C. R. Landis, F. Weinhold, *J. Comput. Chem.* **2013**, *34*, 1429–1437.

[9] T. A. Keith, Todd A. Keith, **2013**.

[10] C. Neese, F.; Wennmohs, F.; Becker, U.; Riplinger, F. Neese, F. Wennmohs, U. Becker, C. Riplinger, *J. Chem. Phys.* **2020**, *152*, 224108.

[11] F. Neese, *Wiley Interdiscip. Rev. Comput. Mol. Sci.* **2018**, *8*, DOI 10.1002/wcms.1327.

[12] F. Neese, F. Wennmohs, A. Hansen, U. Becker, *Chem. Phys.* **2009**, *356*, DOI 10.1016/j.chemphys.2008.10.036.

[13] C. Y. Legault, **2009**.

[14] W. Humphrey, A. Dalke, K. Schulten, *J. Mol. Graph.* **1996**, *14*, 33–38.

[15] C. C. C. and B. DataBase, "CCCBDB listing of precalculated vibrational scaling factors," **2015**.
